# Role of MaABI5-like in abscisic acid-induced cold tolerance of ‘Fenjiao’ banana fruit

**DOI:** 10.1093/hr/uhac130

**Published:** 2022-06-03

**Authors:** Zunyang Song, Xiuhua Lai, Hangcong Chen, Lihua Wang, Xuequn Pang, Yanwei Hao, Wangjin Lu, Weixin Chen, Xiaoyang Zhu, Xueping Li

**Affiliations:** Guangdong Provincial Key Laboratory of Postharvest Science of Fruits and Vegetables/Engineering Research Center for Postharvest Technology of Horticultural Crops in South China, Ministry of Education, College of Horticulture, South China Agricultural University, Guangzhou, Guangdong, 510642, China; Key Laboratory of Food Processing Technology and Quality Control in Shandong Province, College of Food Science and Engineering, Shandong Agricultural University, Tai’an, 271018, China; Guangdong Provincial Key Laboratory of Postharvest Science of Fruits and Vegetables/Engineering Research Center for Postharvest Technology of Horticultural Crops in South China, Ministry of Education, College of Horticulture, South China Agricultural University, Guangzhou, Guangdong, 510642, China; Guangdong Provincial Key Laboratory of Postharvest Science of Fruits and Vegetables/Engineering Research Center for Postharvest Technology of Horticultural Crops in South China, Ministry of Education, College of Horticulture, South China Agricultural University, Guangzhou, Guangdong, 510642, China; Guangdong Provincial Key Laboratory of Postharvest Science of Fruits and Vegetables/Engineering Research Center for Postharvest Technology of Horticultural Crops in South China, Ministry of Education, College of Horticulture, South China Agricultural University, Guangzhou, Guangdong, 510642, China; Guangdong Provincial Key Laboratory of Postharvest Science of Fruits and Vegetables/Engineering Research Center for Postharvest Technology of Horticultural Crops in South China, Ministry of Education, College of Horticulture, South China Agricultural University, Guangzhou, Guangdong, 510642, China; Guangdong Provincial Key Laboratory of Postharvest Science of Fruits and Vegetables/Engineering Research Center for Postharvest Technology of Horticultural Crops in South China, Ministry of Education, College of Horticulture, South China Agricultural University, Guangzhou, Guangdong, 510642, China; Guangdong Provincial Key Laboratory of Postharvest Science of Fruits and Vegetables/Engineering Research Center for Postharvest Technology of Horticultural Crops in South China, Ministry of Education, College of Horticulture, South China Agricultural University, Guangzhou, Guangdong, 510642, China; Guangdong Provincial Key Laboratory of Postharvest Science of Fruits and Vegetables/Engineering Research Center for Postharvest Technology of Horticultural Crops in South China, Ministry of Education, College of Horticulture, South China Agricultural University, Guangzhou, Guangdong, 510642, China; Guangdong Provincial Key Laboratory of Postharvest Science of Fruits and Vegetables/Engineering Research Center for Postharvest Technology of Horticultural Crops in South China, Ministry of Education, College of Horticulture, South China Agricultural University, Guangzhou, Guangdong, 510642, China; Guangdong Provincial Key Laboratory of Postharvest Science of Fruits and Vegetables/Engineering Research Center for Postharvest Technology of Horticultural Crops in South China, Ministry of Education, College of Horticulture, South China Agricultural University, Guangzhou, Guangdong, 510642, China

## Abstract

Abscisic acid (ABA) is a phytohormone essential for plants to respond to various environmental stresses, and abscisic acid-insensitive 5 (ABI5) is a basic leucine zipper transcription factor of the ABA signaling pathway. Exogenous ABA induces cold tolerance in bananas; however, the role of MaABI5-like in ABA-induced cold tolerance remains unexplored. The present study found that exogenous ABA alleviated chilling injury of ‘Fenjiao’ banana, induced the accumulation of endogenous ABA, unsaturated fatty acids, and flavonoid content, and reduced the saturated fatty acid content. Moreover, ABA treatment upregulated the transcription levels of *MaABI5-like*, fatty acid desaturation genes, and flavonoid synthesis-related genes during cold storage. More interestingly, MaABI5-like directly interacted with the promoter of genes related to fatty acid desaturation (*MaFAD3-1*, *MaFAD3-4*, *MaFAD3-5*, *MaFAD6-2*, *MaFAD6-3*) and flavonoid synthesis (*MaPAL-like*, *MaPAL-like1*, *MaC4H-like3*, *Ma4CL-like1*, *Ma4CL-like10*, *MaCHS6-4-like*, and *MaFLS*) and activated their expressions. Furthermore, the transient overexpression of *MaABI5-like* in ‘Fenjiao’ banana fruit and ectopic expression in tomato plants enhanced cold tolerance and upregulated fatty acid desaturation and flavonoid synthesis-related gene transcript levels. The reduced expression of *MaABI5-like* by virus-induced gene silencing in ‘Fenjiao’ banana increased chilling injury and downregulated the expression of fatty acid desaturation and flavonoid synthesis-related genes. Thus, the study indicates that MaABI5-like regulates ABA-induced cold tolerance by increasing unsaturated fatty acid and flavonoid content.

## Introduction

Bananas are the most produced and internationally traded fruits, which are mainly produced in the tropical and subtropical regions [1]. As a typical climacteric fruit, bananas soften and ripen quickly after harvest [2]. The shelf-life and postharvest quality of bananas can be effectively maintained by low-temperature storage [3]. However, bananas are hypersensitive to low temperatures and susceptible to temperatures below 11°C. Consequently, bananas face chilling injury (CI), which causes browning, flavor loss, skin blackening, and ripening disorder [4, 5]. Several studies have closely associated CI with fatty acid and flavonoid contents in banana fruits [5, 6]; however, the mechanism of CI in bananas remains unclear.

Fatty acids constitute a major component influencing plant and fruit resistance to cold stress by maintaining membrane stability and fluidity [6]. Exogenous putrescine treatment increased the content of linolenic acid and linoleic acid in the membrane lipids of kiwifruit fruit and induced tolerance against chilling stress [7]. Salicylic acid treatment significantly alleviated CI in plums [8], pomegranates [9], and loquats [10] during low-temperature storage, and the ratio of unsaturated/saturated fatty acids was significantly higher than that in control samples. Betaine treatment maintained a higher level of unsaturated fatty acids, including linoleic acid and linolenic acid, to reduce the symptoms of cold injury in zucchini fruits [11]. Flavonoids also play an important role in plant and fruit resistance to cold stress [12]. The content of flavonoids in mango fruit with strong cold tolerance was significantly higher than that in fruits sensitive to low temperature [13].

Researchers have reported that transcription factors (TFs), such as MYB transcription factor 4 (MaMYB4) and ethylene responsive factor binding proteins 32 and 33 (CitERF32 and CitERF33), regulate fatty acid and flavonoid contents by interacting with fatty acid desaturation genes and flavonoid synthesis-related genes in fruits. In banana fruits, MaMYB4 regulates the expression of ω-3 fatty acid desaturase genes (*MaFAD3-1/3/4/7*) during the cold stress response [14]. Overexpression of *MdHSFA8a* induced the accumulation of flavonoids in apple plants and enhanced drought tolerance [15]. Meanwhile, CitERF32 and CitERF33 induced flavonoid synthesis by directly binding to the *CitCHIL1* promoter in citrus [16]. However, the role of TFs in regulating fatty acid desaturation and flavonoid synthesis during the cold stress response is largely unknown.

Abscisic acid-insensitive 5 (ABI5) is an abscisic acid (ABA) signaling pathway TF that has been known to regulate seed germination and dormancy, flowering time, growth, and stress adaptation [17, 18]. Numerous ABI5 genes have been studied in various plant species. In apple, MdABI5 controls leaf senescence by interacting with its partners BBX transcription factor 22 (MdBBX22) and WRKY transcription factors (MdWRKY40 and MdbZIP44) [19]. In banana fruits, MaABI5 cooperating with a RING E3 ubiquitin ligase, MaC3HC4-1, regulates ABA-induced cold tolerance [20]. Our previous study found that *MaABI5-like* (XM_009413679.2), which was different from *MaABI5* (XM_009395750) reported by Chen *et al*. [20], was induced by exogenous ABA treatment [21]. Although several ABI5 genes have been studied in plants, their mechanism in the cold stress response in fruits is not clear.

‘Fenjiao’ (*Musa* spp. ABB Pisang Awak) is a popular banana cultivar widely cultivated in the south of China. It is resistant to environmental stress and highly nutritious [22]. However, ‘Fenjiao’ banana fruits ripen and deteriorate rapidly after harvest, which significantly limits their storage and transport compared with other commercial cultivars [23]; therefore, low-temperature storage has been used as a standard technology for storage and transportation. However, ‘Fenjiao’ banana fruits are hypersensitive to low temperature, which leads to CI at temperatures under 11°C [21]. Cold stress causes severe CI symptoms in ‘Fenjiao’ banana, including peel browning and softening disorder [5, 21]. Our previous study found that MaABI5-like promotes starch and cell wall degradation by directly activating the transcription activity of genes related to starch and cell wall degradation, but cold stress causes softening disorder by repressing the expression of starch and cell wall degradation genes and *MaABI5-like* in ‘Fenjiao’ banana [21]. However, the mechanism by which MaABI5-like regulates cold stress response has not been revealed yet.

The present study investigates the possible mechanism of ABA-induced chilling stress resistance in ‘Fenjiao’ banana. ABA treatment mainly induced endogenous ABA, unsaturated fatty acid, and flavonoid contents by upregulating *MaABI5-like* expression. The roles of *MaABI5-like* in the cold stress response were validated by transient and ectopic overexpression of *MaABI5-like* in ‘Fenjiao’ banana and tomato plants. This study provides new insights into the regulatory network underlying ABA-induced cold tolerance in the economically important banana.

## Results

### Exogenous abscisic acid treatment enhanced cold tolerance of ‘Fenjiao’ banana fruit

**Figure 1:**
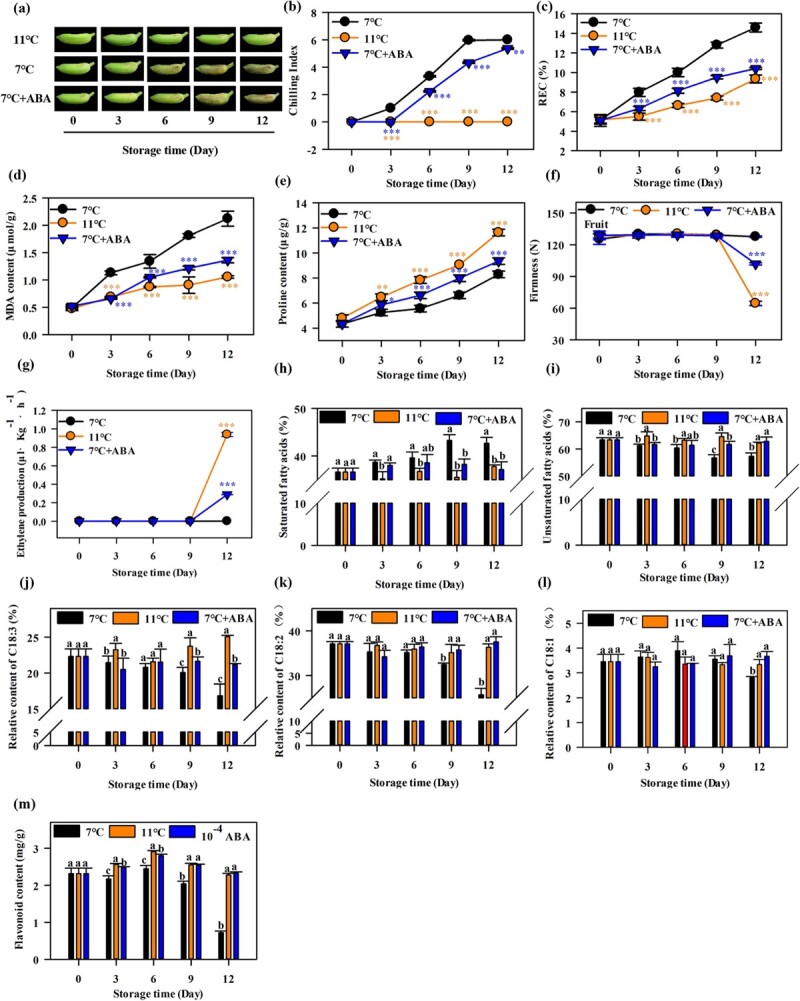
Exogenous ABA alleviates CI symptoms caused by 7°C treatment in ‘Fenjiao’ banana fruit. **a** Fruit ripening process under 11°C, 7°C and 7°C + ABA treatments. **b**–**g** Changes in chilling index (**b**), REC (**c**) MDA content (**d**), proline content (**e**), fruit firmness (**f**), and ethylene production (**g**) in ABA-treated ‘Fenjiao’ fruit pulp during storage at 7°C and control ‘Fenjiao’ fruit pulp during storage at 7°C and 11°C. ^*^P < .05, ^**^P < .01, ^***^P < .001. **h**–**m** Changes in the contents of saturated fatty acids (**h**), unsaturated fatty acids (**i**), C18:3 (**j**), C18:2 (k), C18:1 (**l**), and flavonoids (**m**) in ABA-treated ‘Fenjiao’ fruit pulp during storage at 7°C and control ‘Fenjiao’ fruit pulp during storage at 7°C and 11°C. Different letters indicate significantly different values (*P* < .05).

In ‘Fenjiao’ banana, CI symptoms, such as brown patches and pitting, began to appear after 3 days of storage at 7°C (Fig. 1a); the CI index increased with storage time (Fig. 1b). No CI symptom was observed for ‘Fenjiao’ bananas stored at 11°C. Meanwhile, ABA treatment (10^−4^ M) relieved the CI symptoms and repressed the increase in CI index under 7°C storage (Fig. 1a and b). The relative conductivity (REC) and malondialdehyde (MDA) content increased with storage in all groups (7°C, 11°C, and 7°C + ABA). ABA treatment reduced the REC and MDA content, with significantly lower REC and MDA content observed in ABA-treated fruit than in control fruit under storage at 7°C (Fig. 1c and d). However, the REC and MDA content of the ‘Fenjiao’ banana fruits treated with ABA was higher compared with fruits stored at 11°C (Fig. 1c and d). The proline content also increased in all groups with storage. The proline content under ABA treatment was higher than that under 7°C and lower than that under 11°C, consistent with the fruit CI symptoms (Fig. 1e). Fruit firmness remained stable and maintained a high level for fruit stored at 7°C during the whole storage period. For 11°C storage and ABA-treated fruit, fruit firmness started decreasing from the ninth day, and ABA-treated fruits showed a higher level of firmness than fruits stored at 11°C (Fig. 1f). Ethylene production was not detected during the whole storage period for fruits stored at 7°C. For 11°C storage and ABA treatment, ethylene was detected from the ninth day and increased gradually, and higher production was observed for 11°C storage than ABA treatment (Fig. 1g). These results indicate that ABA treatment enhanced cold tolerance and induced ethylene production in ‘Fenjiao’ banana fruits under cold storage.

**Figure 2:**
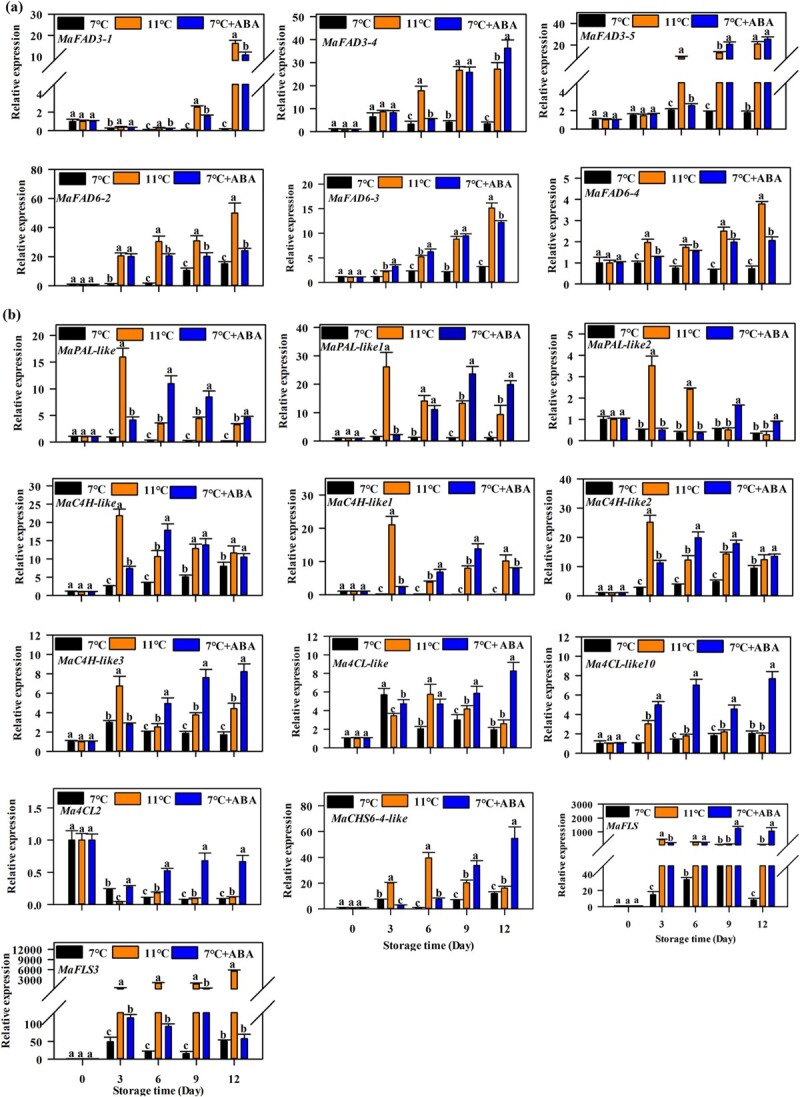
Effects of ABA treatments on transcript levels of fatty acid desaturation and flavonoid synthesis related genes in ‘Fenjiao’ banana pulp. **a**, **b** Transcript levels of fatty acid desaturation genes (**a**) and flavonoid synthesis-related (**b**) genes. The transcription of each gene at different points are relative to day 0 set as 1. Different letters indicate significantly different values (*P* < .05).

### Exogenous abscisic acid increased unsaturated fatty acid and flavonoid contents of ‘Fenjiao’ banana fruits

The contents of fatty acids and flavonoids are closely related to the cold tolerance of fruits [24]. As shown in Fig. 1h–m, the content of saturated fatty acids gradually increased during storage under chilling temperature (7°C). Cold stress induced the accumulation of saturated fatty acids and reduced the content of unsaturated fatty acids, such as linolenic acid (18:3), linoleic acid (18:2), and oleic acid (18:1), and flavonoids compared with 11°C storage. Meanwhile, ABA treatment significantly reduced the content of saturated fatty acids and increased the content of unsaturated fatty acids compared with 7°C treatment; however, the content of saturated fatty acids was higher than at 11°C, but contents of the unsaturated fatty acid oleic acid (18:1) and flavonoids were lower.

**Figure 3:**
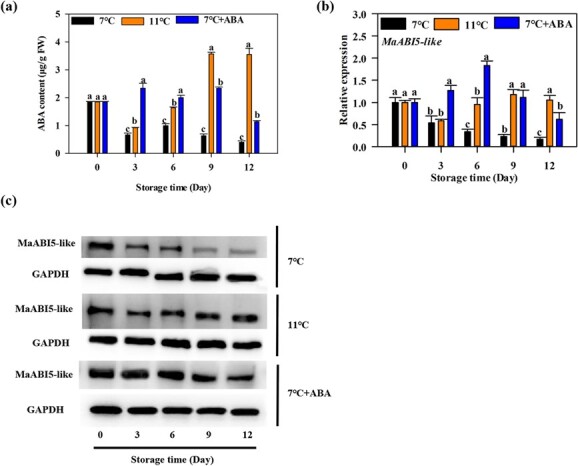
Endogenous ABA content and expression of *MaABI5-like* in pulp during ‘Fenjiao’ fruit storage. **a** Content of endogenous ABA in ‘Fenjiao’ fruits under different storage conditions. Different letters indicate significantly different values (*P* < .05). **b** Expression of *MaABI5-like* in pulp during ‘Fenjiao’ fruit storage. The expression levels of each gene at different points are relative to day 0 set as 1. Different letters indicate significantly different values (*P* < .05). **c** Protein level of MaABI5-like in pulp during ‘Fenjiao’ fruit storage.

### Exogenous abscisic acid induced transcription of genes related to fatty acid desaturation and flavonoid synthesis

RNA-Seq revealed a few differentially expressed genes (DEGs) related to fatty acid desaturation and flavonoid synthesis in ‘Fenjiao’ bananas during cold storage (Supplementary Data Fig. S1). Fourteen fatty acid desaturation genes and 22 flavonoid synthesis-related genes were found. The transcription of six fatty acid desaturation genes (*MaFAD3-1*, *MaFAD3-4*, *MaFAD3-5*, *MaFAD6-2*, *MaFAD6-3,* and *MaFAD6-4*) and 13 flavonoid synthesis-related genes (*MaPAL-like*, *MaPAL-like1*, *MaPAL-like2*, *MaC4H-like*, *MaC4H-like1*, *MaC4H-like2*, *MaC4H-like3*, *Ma4CL-like1*, *Ma4CL-like10*, *Ma4CL2*, *MaCHS6-4-like*, *MaFLS*, and *MaFLS3*) was higher under storage at 11°C than 7°C. However, ABA treatment significantly induced the expression levels of these genes (Fig. 2, Supplementary Data Fig. S1). These observations indicate that the transcription of these genes was closely related to the contents of fatty acids and flavonoids and fruit CI.

### MaABI5-like targets ABRE/G-box element of the promoters of fatty acid desaturation genes and flavonoid synthesis-related genes

Furthermore, to determine whether chilling stress during storage affected the endogenous ABA levels, the ABA content of fruits was quantified by liquid chromatography–mass spectrometry in control and ABA-treated banana fruit pulp. As shown in Fig. 3a, the content of ABA in fruits under 11°C storage decreased on day 3 and then increased with storage. Meanwhile, 7°C storage significantly reduced the content of ABA compared with 11°C. ABA treatment induced endogenous ABA content compared with 7°C treatment. In addition, ABA treatment showed higher ABA content than 11°C treatment on day 3 and day 6, but lower content on day 9 and day 12. Cold stress (7°C) also significantly inhibited the expression of ABA synthesis genes (*MaZEP*, *MaZEP2*, *MaZEP*2) and signal transduction-related genes (*MaPYL4*, *MaPYL4-like*, *MaPP2C-like*, *MaPP2C-like1*, and *MaABI5-like*), and promoted the expression of ABA degradation genes (*MaABAH1-like*, *MaABAH3-like*) (Supplementary Data Fig. S2). These data suggest that exogenous ABA induced endogenous ABA during chilling storage.

ABI5 is an important transcription factor of the ABA signaling pathway, and it regulates the target genes by directly interacting with the ABRE/G-box element in the promoter of these target genes [25]. Our previous study found that cold stress (7°C) repressed MaABI5-like gene and protein expressions [21]. As shown in Fig. 3b and c, 7°C storage severely inhibited MaABI5-like transcript and protein levels compared with 11°C treatment, consistent with the previous studies [21]. ABA treatment significantly induced the transcript and protein levels of MaABI5-like compared with 7°C treatment, to a level higher than that at 11°C (Fig. 3b and c).

**Figure 4:**
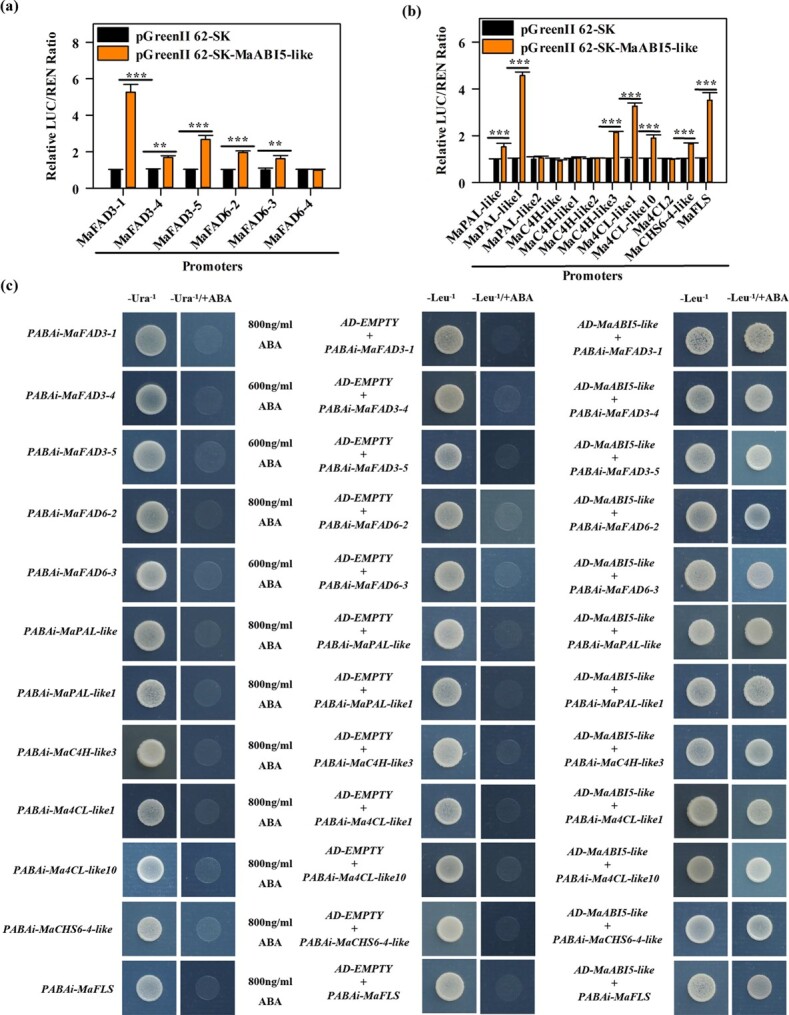
MaABI5-like activates the expression of genes related to fatty acid desaturation and flavonoid synthesis. **a**, **b** Transcriptional activity of MaABI5-like on the promoters of fatty acid desaturation (**a**) and flavonoid synthesis (**b**) genes. Empty vector was employed as a calibrator (set as 1). ^**^P < .01, ^***^P < .001. **c** Yeast growth assays showing the interaction between MaABI5-like and the five fatty acid desaturation and seven flavonoid synthesis genes. The transformed yeast cells were grown on SD medium with AbA but without Leu.

Sequence analysis revealed the presence of the ABRE/G-box element in the promoter region of six fatty acid desaturation genes (*MaFAD3-1*, *MaFAD3-4*, *MaFAD3-5*, *MaFAD6-2*, *MaFAD6-3*, and *MaFAD6-4*) and 12 flavonoid synthesis-related genes (*MaPAL-like*, *MaPAL-like1*, *MaPAL-like2*, *MaC4H-like*, *MaC4H-like1*, *MaC4H-like2*, *MaC4H-like3*, *Ma4CL-like1*, *Ma4CL-like10*, *Ma4CL2*, *MaCHS6-4-like*, and *MaFLS*) (Supplementary Data Fig. S3), indicating the role of MaABI5-like in regulating these genes. Further, to investigate this, their promoters were inserted in pGreenII 0800-LUC, and the full coding sequence of *MaABI5-like* was inserted in the pGreenII 62-SK vector for the dual-luciferase assay (Supplementary Data Fig. S4a). As shown in Fig. 4a and b, the activities of *MaFAD3-1*, *MaFAD3-4*, *MaFAD3-5*, *MaFAD6-2*, *MaFAD6-3*, *MaPAL-like*, *MaPAL-like1*, *MaC4H-like3*, *Ma4CL-like1*, *Ma4CL-like10*, *MaCHS6-4-like*, and *MaFLS* promoters were dramatically upregulated in the presence of MaABI5-like compared with the control.

**Figure 5:**
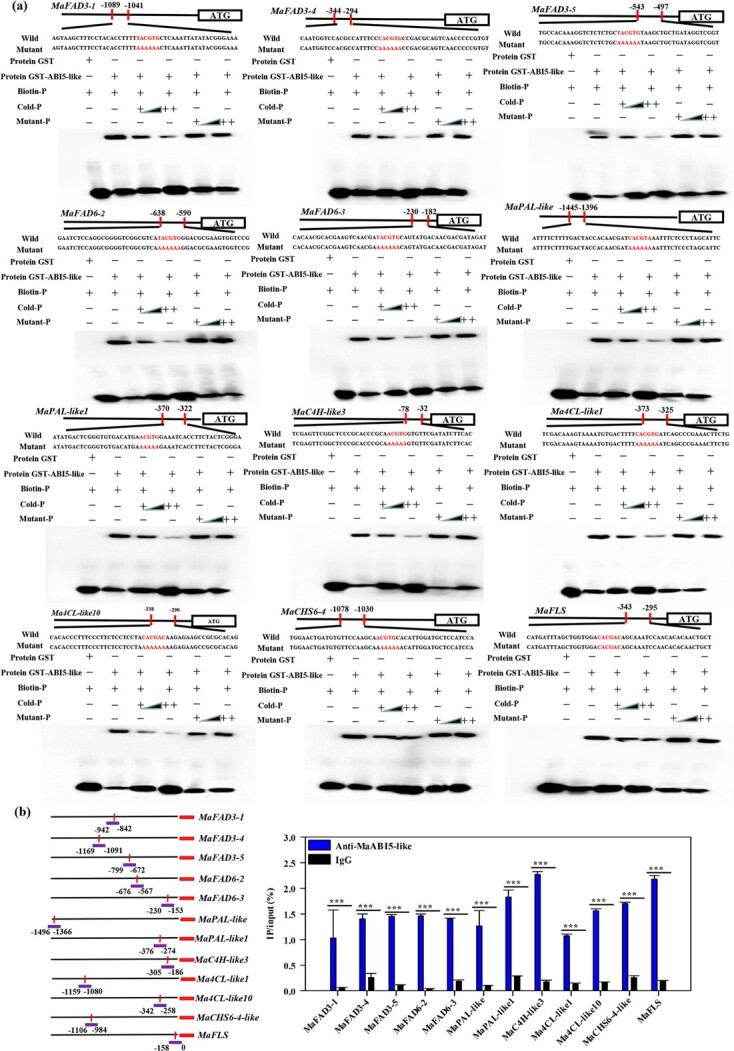
MaABI5-like binds to the promoters of five fatty acid desaturation and seven flavonoid synthesis genes *in vitro* and *in vivo*. **a** Binding of MaABI5-like with the promoters of five fatty acid desaturation and seven flavonoid synthesis genes in EMSA assays. The GST-MaABI5-like was mixed with the probes of five fatty acid desaturation and seven flavonoid synthesis gene promoters, including the ABRE/G-box binding site and the mutant probe AAAAA, which is shown with red letters. ‘+’ represents presence, ‘−’ represents absence, ‘++’ represents increasing amounts of probe. **b** (Left) Promoter structure of the five fatty acid desaturation and seven flavonoid synthesis genes. The red lines indicate the *cis*-elements of ABI5-like. (Right) ChIP–qPCR assay of binding of MaABI5-like to the promoter of five fatty acid desaturation and seven flavonoid synthesis genes. Values are the percentage of DNA fragments co-immunoprecipitated with anti-ABI5-like antibodies or the non-specific antibody (anti-IgG) relative to the input DNA. ^***^P < .001.

To further verify whether MaABI5-like directly binds to the promoter of *MaFAD3-1*, *MaFAD3-4*, *MaFAD3-5*, *MaFAD6-2*, *MaFAD6-3*, *MaPAL-like*, *MaPAL-like1*, *MaC4H-like3*, *Ma4CL-like1*, *Ma4CL-like10*, *MaCHS6-4-like*, and *MaFLS*, a yeast one-hybrid (Y1H) assay was conducted. As shown in Fig. 4c, MaABI5-like interacted with the promoters of these fatty acid desaturation genes and flavonoid synthesis-related genes in yeast. The electrophoretic mobility shift assay (EMSA) showed that MaABI5-like directly bound with the promoters of *MaFAD3-1*, *MaFAD3-4*, *MaFAD3-5*, *MaFAD6-2*, *MaFAD6-3*, *MaPAL-like*, *MaPAL-like1*, *MaC4H-like3*, *Ma4CL-like1*, *Ma4CL-like10*, *MaCHS6-4-like*, and *MaFLS* containing the ABRE/G-box (Fig. 5a). Chromatin immunoprecipitation (ChIP)–qPCR analysis also indicated that the promoters of *MaFAD3-1*, *MaFAD3-4*, *MaFAD3-5*, *MaFAD6-2*, *MaFAD6-3*, *MaPAL-like*, *MaPAL-like1*, *MaC4H-like3*, *Ma4CL-like1*, *Ma4CL-like10*, *MaCHS6-4-like*, and *MaFLS* containing the ABRE/G-box element were significantly enriched by anti-MaABI5-like compared with the negative control IgG (Fig. 5b). Overall, these results indicate that MaABI5-like acts as a positive transcriptional activator of a subset of these fatty acid desaturation genes and flavonoid synthesis-related genes by directly interacting with the ABRE/G-box motif of their promoters.

### Transient overexpression of *MaABI5-like* enhanced the cold tolerance of ‘Fenjiao’ banana fruit

**Figure 6:**
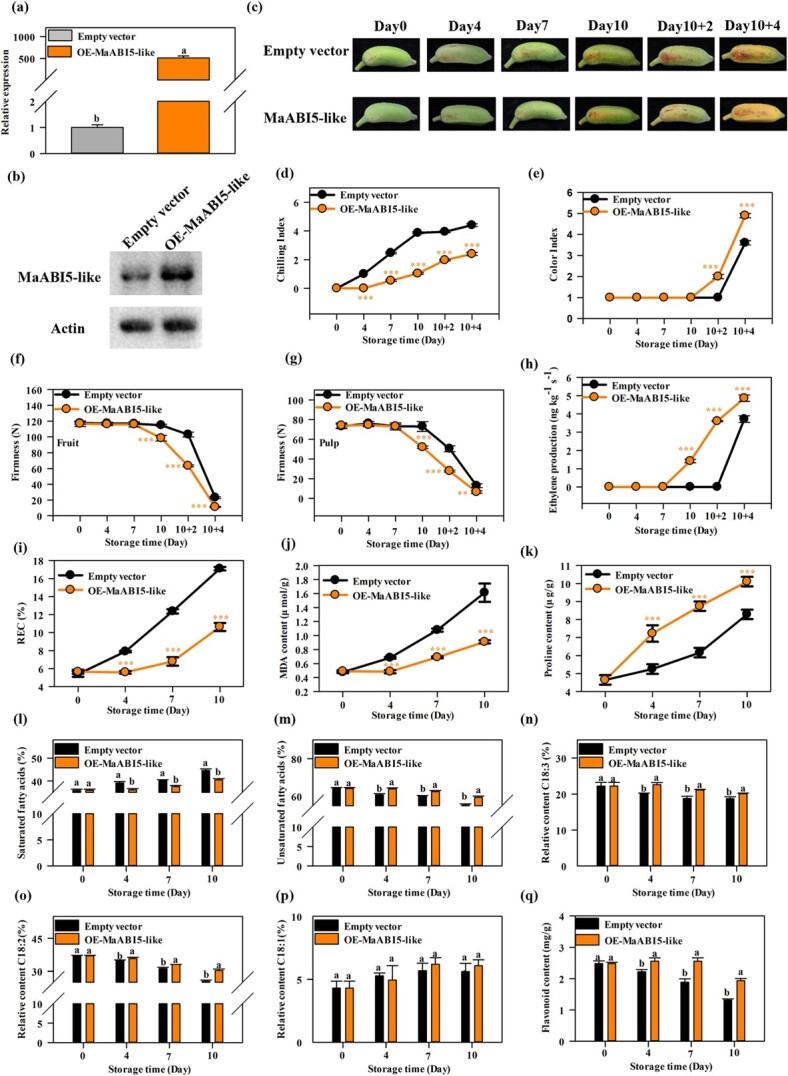
Overexpression of *MaABI5-like* in ‘Fenjiao’ banana improves tolerance to cold stress. **a**, **b** Transcript (**a**) and protein (**b**) levels of MaABI5-like in ‘Fenjiao’ fruit pulp of empty vector and *MaABI5-like*-overexpressing fruits was determined by RT–qPCR and western blotting. **c** ‘Fenjiao’ fruit cold stress storage and ripening process of empty vector and *MaABI5-like*-overexpressing line. **d**–**h** Changes in CI (**d**), color index (**e**), firmness of whole fruit (**f**) and pulp (**g**), and ethylene production (**h**) during cold stress storage and fruit ripening. **i**–**k** Changes in REC (**i**), MDA content (**j**), and proline content (**k**) during cold stress storage. ^**^P < .01, ^***^P < .001. **l**–**q** Changes in contents of saturated fatty acids (**l**), unsaturated fatty acids (**m**), C18:3 (**n**), C18:2 (**o**), C18:1 (**p**), and flavonoids (**q**) in OE-*MaABI5-like* and empty control vector ‘Fenjiao’ fruit pulp during storage at 7°C. Different letters indicate significantly different values (*P* < .05).

Further, *MaABI5-like* was transiently overexpressed in ‘Fenjiao’ fruit to investigate their role in cold tolerance. The dramatic increase in *MaABI5-like* gene and protein expression levels confirmed that *MaABI5-like* was overexpressed successfully in ‘Fenjiao’ banana (Fig. 6a and b). Significant differences in CI were observed between fruits with the empty vector and fruits overexpressing the *MaABI5-like* construct (Fig. 6c and d). CI symptoms were slightly visible on day 4 in empty vector control fruit, whereas no CI was observed in the *MaABI5-like-*overexpressing fruit on day 4 (Fig. 6c and d). These CI symptoms increased with fruit storage and appeared more severe after ripening under normal temperature in the empty vector control fruit (Fig. 6c and d). The *MaABI5-like-*overexpressing fruit showed significantly lower CI than the control during the entire storage and ripening period. The *MaABI5-like*-overexpressing fruit showed accelerated fruit ripening characteristics, including fruit color index, firmness, and ethylene production, compared with the empty control fruit (Fig. 6e–h). REC, MDA content, and proline content increased with storage during chilling storage in both groups (Fig. 6i–k). Meanwhile, REC and MDA content were significantly repressed in *MaABI5-like*-overexpressing fruit compared with empty vector fruit, while the proline content was dramatically induced (Fig. 6i–k). Overexpression of *MaABI5-like* increased the unsaturated fatty acid and flavonoid contents. The results showed that *MaABI5-like-*overexpressing banana fruit showed significantly higher levels of unsaturated fatty acids and flavonoids but lower saturated fatty acid compared with the empty control (Fig. 6l–q). Moreover, *MaABI5-like* overexpression induced transcript levels of *MaFAD3-1*, *MaFAD3-4*, *MaFAD3-5*, *MaFAD6-2*, *MaFAD6-3*, *MaPAL-like*, *MaPAL-like1*, *MaC4H-like3*, *Ma4CL-like1*, *Ma4CL-like10*, *MaCHS6-4-like*, and *MaFLS* compared with the empty control (Supplementary Data Fig. S5)*.*

### Reduced expression of *MaABI5-like* enhanced chilling injury of ‘Fenjiao’ banana fruit

**Figure 7:**
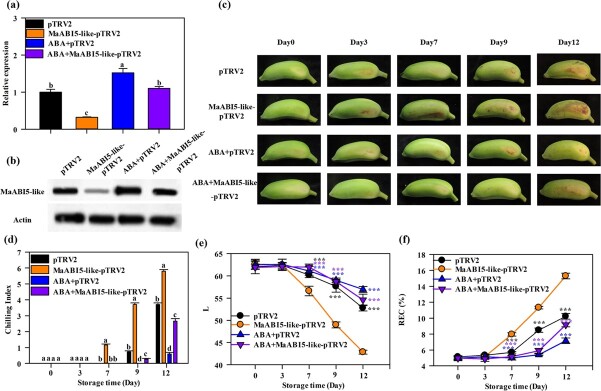
Reduced expression of *MaABI5-like* enhanced the CI of ‘Fenjiao’ fruits. **a**, **b** Transcript (**a**) and protein (**b**) levels of *MaABI5-like* in ‘Fenjiao’ fruit pulp of pTRV2, MaABI5-like-pTRV2, ABA+pTRV2 and ABA+MaABI5-like-pTRV2 were determined by RT–qPCR and western blotting. **c** Photographs of ‘Fenjiao’ fruit under cold stress storage without (pTRV2, MaABI5-like-pTRV2) and with (ABA+pTRV2, ABA+MaABI5-like-pTRV2) ABA treatment. **d**–**h** Changes in chilling index (**d**), L value (**e**) and REC (**f**) during cold stress storage. ^***^P < .001. Different letters indicate significantly different values (*P* < .05).

To further examine the possible function of MaABI5-like in cold tolerance, *MaABI5-like* was transiently silenced in ‘Fenjiao’ fruits using virus-induced gene silencing (VIGS). As shown in Fig. 7, compared with the pTRV2-empty vector control, significant decreases in *MaABI5-like* gene and protein expression levels were observed in silenced fruits (Fig. 7a and b). Significant differences in CI were observed between the silenced fruits and the empty vector control. CI symptoms were slightly visible on day 7 in *MaABI5-like-*silenced fruits, whereas no CI was observed in the pTRV2-empty control fruits. These CI symptoms increased with fruit storage (Fig. 7c and d). The *MaABI5-like-*silenced fruits showed significantly higher CI than pTRV2-empty control fruits during the whole storage period (Fig. 7c and d). Exogenous ABA treatment induced the expression of *MaABI5-like* transcript and protein levels both in the pTRV2-empty control and MaABI5-like-pTRV2 fruits, and alleviated the CI symptoms compared with no ABA treatment (Fig. 7a–d). Notably, the MaABI5-like-pTRV2 fruits showed more severe CI symptoms than the pTRV2-empty control after ABA treatment, but less severe symptoms than fruits without ABA treatment (Fig. 7c and d). These results indicated that exogenous ABA enhancement of cold tolerance depended on ABI5-like protein. The L value, which indicating fruit lightness, decreased with storage in all groups, the *MaABI5-like-*silenced fruits showed a lower L value than pTRV2-empty, ABA+pTRV2-empty, and ABA+MaABI5-like-PTRV2 fruits, and the L value of ABA+MaABI5-like-pTRV2 was lower than that of ABA+pTRV2 fruits and higher than that of *MaABI5-like-*silenced fruits (Fig. 7e).

**Figure 8:**
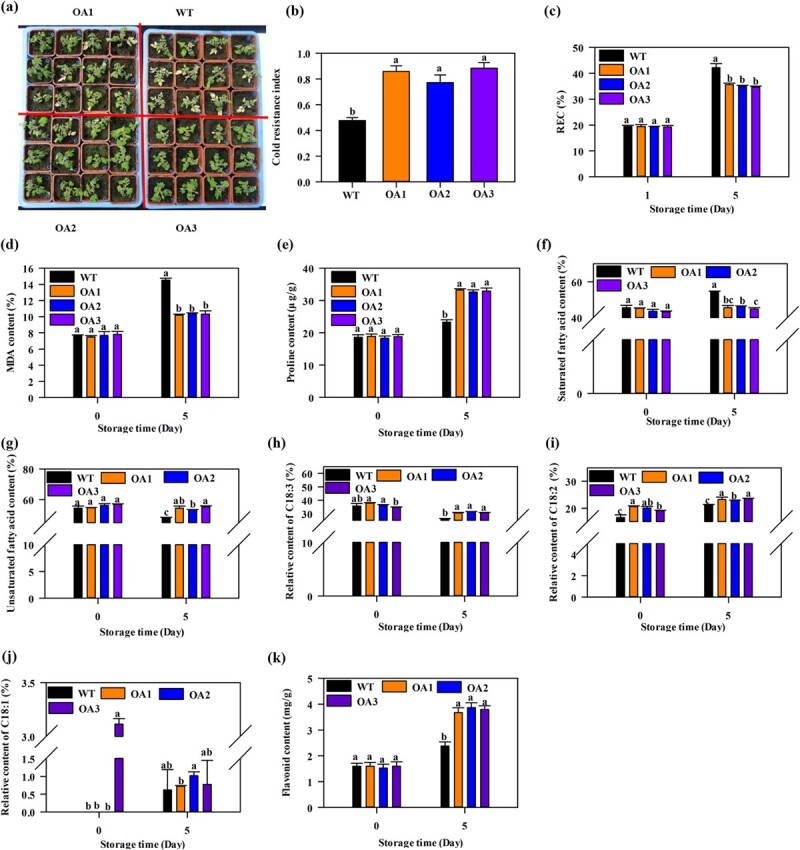
Overexpression of *MaABI5-like* in tomato improves tolerance to cold stress. **a** Phenotype differences between WT and transgenic plants under 4°C cold treatment for 5 days. **b**–**e** Changes in chilling index (**b**), REC (**c**), MDA content (**d**), and proline content (**e**) in *MaABI5-like*-overexpressing plants and WT tomato plants during cold storage. **f**–**k** Changes in the content of saturated fatty acids (**f**), unsaturated fatty acids (**g**), C18:3 (**h**), C18:2 (**i**), C18:1 (**j**), and flavonoids (**k**) in *MaABI5-like*-overexpression and WT tomato plant during cold storage. Different letters indicate significantly different values (*P* < .05).

The REC increased with storage during chilling storage in all groups. Meanwhile, REC was significantly induced in *MaABI5-like*-silenced fruits compared with the pTRV2-empty control. The REC of ABA+MaABI5-like-pTRV2 fruits was higher than that of ABA+pTRV2 fruits and lower than that of *MaABI5-like-*silenced fruits (Fig. 7f). Additionally, *MaABI5-like-*silenced fruits showed reduced expression levels of *MaFAD3-1*, *MaFAD3-4*, *MaFAD3-5*, *MaFAD6-2*, *MaFAD6-3*, *MaPAL-like*, *MaPAL-like1*, *MaC4H-like3*, *Ma4CL-like1*, *Ma4CL-like10*, *MaCHS6-4-like*, and *MaFLS* compared with the pTRV2-empty control, and ABA treatment induced their expression in both pTRV2-empty and silenced fruits. Transcript levels of these genes were lower in MaABI5-like-pTRV2 fruits than in pTRV2 fruits (Supplementary Data Fig. S6)*.* All of these results indicated that *MaABI5-like* silencing enhanced CI and enhancement of cold tolerance by exogenous ABA treatment depended on MaABI5-like protein.

### Ectopic overexpression of *MaABI5-like* enhanced cold tolerance in tomato plant

Finally, the role of MaABI5-like in the chilling stress response was investigated by stable overexpression of the gene in tomatoes. Three independent transgenic lines (OA1, OA2, and OA3) with a high expression level of *MaABI5-like*, constructed in our previous work, were selected for investigation [21]. Four-week-old wild-type (WT) and *MaABI5-like-*overexpressing plants were transferred to a 4°C chamber for 1 week. Under normal growth circumstances, plants grew well, and the difference in growth phenotype and physiological indexes between the WT and transgenic plants was non-significant. After 5 days of chilling stress (4°C), a difference was observed in cold tolerance between *MaABI5-like*-overexpressing lines and WT; *MaABI5-like*-overexpressing lines showed a higher tolerance index than WT (Fig. 8a and b). Meanwhile, visible leaf withering was less severe in the transgenic lines than in the WT plants after chilling stress (4°C) for 5 days. Chilling stress (4°C) increased REC and the MDA and proline contents in all plants. The REC and MDA content were severely repressed in *MaABI5-like*-overexpressing lines (Fig. 8c and d), while the proline content was induced compared with WT (Fig. 8e). The content of saturated fatty acids was reduced, and the content of unsaturated fatty acids [linolenic acid (18:3), linoleic acid (18:2)] and flavonoids was induced in *MaABI5-like*-overexpressing lines compared with WT under chilling stress (4°C) for 5 days (Fig. 8f–i, k); however, no significant difference was observed in oleic acid (18:1) (Fig. 8j). Moreover, the transcript levels of *SlFAD3-1*, *SlFAD3-4*, *SlFAD3-5*, *SlFAD6-2*, *SlFAD6-3*, *SlPAL-like*, *SlPAL-like1*, *SlC4H-like3*, *Sl4CL-like1*, *Sl4CL-like10*, *SlCHS6-4-like*, and *SlFLS* increased in *MaABI5-like*-overexpressing lines compared with WT under chilling stress (4°C) for 5 days (Supplementary Data Fig. S7). However, no significant difference between the *MaABI5-like*-overexpressing lines and WT was observed after storage under the chilling condition in tomato fruit (Supplementary Data Fig. S8).

## Discussion

Chilling injury is a physiological disorder that significantly reduces the postharvest quality of tropical and subtropical fruits and vegetables [26]. ‘Fenjiao’ banana is a cold-sensitive fruit, which suffers CI when exposed to temperatures below 11°C [5]. ‘Fenjiao’ banana suffered CI and softening disorder when stored at 7°C for 12 days [21]. The present study found that cold stress (7°C) caused CI, significantly inhibited the expression of ABA synthesis genes and signal transduction-related genes, and promoted the expression ABA degradation genes (Supplementary Data Fig. S2), while ABA treatment induced cold tolerance and alleviated CI symptoms of ‘Fenjiao’ bananas (Fig. 1). Typically, the cold stress response of plants and fruits is a complex process involving different metabolic pathways and multiple gene regulations [27]. Specifically, the composition of fatty acids is associated with cold tolerance. For example, exogenous oxalic acid [28], putrescine [7], salicylic acid [9], and glycine betaine [11] increased the ratio of unsaturated fatty acids/saturated fatty acids and induced fruit cold tolerance. In this study, cold stress (7°C) significantly reduced the content of unsaturated fatty acids and increased the content of saturated fatty acids. Meanwhile, exogenous ABA treatment increased the content of unsaturated fatty acids and reduced the content of saturated fatty acids (Fig. 1). Flavonoids are also important substances that play a key role in cold stress resistance, due to their antioxidant activity [29]. Sudheeran *et al*. [13] found that the content of flavonoids in mango fruit with strong cold tolerance was significantly higher than that in mango fruits sensitive to low temperature; flavonols reduced ROS in response to cold storage, resulting in reduced lipid peroxidation and reduced chilling injuries. In plum fruits, phenylalanine treatment significantly alleviated CI by inducing antioxidant capacity and increasing the flavonoid content [30]. Meanwhile, the combined use of chitosan and potassium sorbate increased the flavonoid content and enhanced the cold tolerance of pomegranate fruits by inducing the activity of phenylalanine ammonia-lyase (PAL) and repressing the activity of polyphenol oxidase (PPO) [31]. In strawberry, an edible coating of chia seed mucilage/bacterial cellulose alleviated CI by maintaining the content of flavonoids and antioxidant activity [32]. Our results showed that cold stress (7°C) severely reduced the content of flavonoids and increased the content of MDA (indicating the membrane lipid peroxidation index), but ABA treatment increased the content of flavonoids and reduced the content of MDA. All this information indicated that flavonoids functioned as important non-enzymatic antioxidants, which could help to improve the antioxidant systems of the plant and reduce ROS damage, resulting in enhanced chilling tolerance. These results are in agreement with the chilling tolerance phenotype. Our findings indicate that ABA treatment alleviated CI in ‘Fenjiao’ bananas via fatty acid desaturation and flavonoid synthesis. RNA-Seq and RT–qPCR analysis showed that cold stress (7°C) inhibited 6 fatty acid desaturation genes and 13 flavonoid synthesis-related genes, but ABA treatment upregulated the transcript levels of these genes (Fig. 2, Supplementary Data Fig. S1). Moreover, ABA treatment induced ethylene production compared with 7°C treatment (Fig. 1g), which is similar to our previous study, in which exogenous ABA treatment significantly relieved CI symptoms and promoted fruit ripening [21].

ABA is a vital phytohormone involved in regulating environment stress responses and fruit ripening [33]. Several studies have shown that cold stress reduces ABA accumulation. In citrus fruit, low-temperature storage significantly reduced the ABA content compared with the ambient temperature during postharvest storage [34]. Low temperature reduced ABA accumulation during grape development [35]. In peach fruit, cold stress decreases ABA content and causes CI of fruits, and exogenous ABA enhanced fruit cold tolerance by reduced the decrease in ABA content [36, 37]. In *Gladiolus hybridus*, cold stress severely decreased the ABA content [38]. However, several studies have also shown that exogenous ABA treatment and cold stress induce the content of endogenous ABA. In *Elymus nutans* [39] and banana [40], cold storage increased the content of ABA, and exogenous ABA treatment induced ABA accumulation. The present work found that the ABA content significantly decreased under chilling storage and was induced by exogenous ABA treatment, consistent with our previous study [21]. These results prove that ABA plays a vital role in the cold stress response in banana fruits.

TFs play an important role in regulating various plant physiological processes, such as nitrogen metabolism [41], CI [40], and fruit ripening [5, 42]. They also act in the signaling of plant hormones, such as ethylene [43], cytokinin [44], and auxin [45]. Our previous study found that chilling stress severely repressed the transcription of *MaABI5-like*, which plays an important role in fruit softening and regulates softening disorder under chilling stress [21]. However, the molecular mechanism of MaABI5-like in CI remission is still not clear. In this study, cold stress (7°C) inhibited the expression of *MaABI5-like*, while ABA treatment induced the expression (Fig. 3). The result is consistent with the trend observed for OsABI5, which was induced by salt and ABA, but repressed by cold and drought stress in rice [46]. In *Gladiolus hybridus*, cold stress significantly repressed the expression of *GhABI5* during 4°C storage [38]. Meanwhile, *CsWRKY46* overexpression resulted in higher seedling survival rates and *AtABI5* expression upon freezing treatment in *Arabidopsis* compared with WT [47]. ArCspA (cold-shock protein) transgenics showed improved cold tolerance and upregulated expression of *OsABI5* in rice plants [48], indicating the role of MaABI5-like in regulating CI of ‘Fenjiao’ banana.

Our results also revealed *MaABI5-like* expression patterns consistent with the transcript levels of fatty acid desaturation genes and flavonoid synthesis-related genes. Several studies have reported that TFs regulating fatty acid desaturation and flavonoid synthesis regulate the cold stress response also. For example, MaMYB4 regulated the expression of *MaFAD3-1*/*3*/*4/7* in banana fruit during the cold stress response [14]. In citrus fruits, CitERF32 and CitERF33 induced flavonoid synthesis by regulating the expression of *CitCHIL1* [16]. MdHSFA8a induced flavonoid synthesis and improved drought tolerance in the apple [15]. Moreover, ABI5 is a b-ZIP transcription factor, which regulates various metabolic pathways of plants and fruits. MdABI5 interacts with its partners to regulate ABA-mediated leaf senescence in the apple [19]. MaABI5 regulates ABA-induced cold tolerance by cooperating with a RING E3 ubiquitin ligase, MaC3HC4-1 in banana fruit [20]. Meanwhile, herein we demonstrate that MaABI5-like participates in ABA-induced cold tolerance by upregulating the expression of fatty acid desaturation genes and flavonoid synthesis-related genes, including *MaFAD3-1*, *MaFAD3-4*, *MaFAD3-5*, *MaFAD6-2*, *MaFAD6-3*, *MaPAL-like*, *MaPAL-like1*, *MaC4H-like3*, *Ma4CL-like1*, *Ma4CL-like10*, *MaCHS6-4-like*, and *MaFLS*, by binding to its promoters (Figs 4 and 5). Moreover, our previous study found that MaABI5-like interacted with MaEBF1. The present study found that this interaction enhanced the expression of a few fatty acid desaturation genes and flavonoid synthesis-related genes (*MaFAD3-4*, *MaFAD3-5*, *MaFAD6-3*, *MaPAL-like*, *MaC4H-like3*, *Ma4CL-like10*, and *MaCHS6-4-like*) (Supplementary Data Fig. S4b). Additionally, the transient and ectopic overexpression of *MaABI5-like* in ‘Fenjiao’ banana and the tomato plant induced cold tolerance and enhanced the transcript levels of fatty acid desaturation and flavonoid synthesis-related genes (Figs 6 and 8). Meanwhile, *TaABI5* overexpression improved the survival rate of tobacco (*Nicotiana tabacum*) under freezing temperatures [49]. Our results are consistent with previous studies that showed ABI5-like’s positive role in cold tolerance.

To conclude, we propose a model explaining the possible molecular mechanism of ABA-induced cold tolerance in ‘Fenjiao’ banana fruits (Supplementary Data Fig. S9). Under no chilling, low-temperature storage, MaABI5-like activates the transcription of fatty acid desaturation genes and flavonoid synthesis-related genes. However, under chilling temperature the ABA content and the MaABI5-like accumulation gets repressed, which further represses the expression of fatty acid desaturation and flavonoid synthesis-related genes. Furthermore, exogenous ABA treatment induces endogenous ABA content and MaABI5-like protein accumulation, activating the expression of genes related to fatty acid desaturation and flavonoid synthesis, resulting in enhanced chilling tolerance of banana fruits.

## Materials and methods

### Plant materials and treatments

Mature-green ‘Fenjiao’ banana (*Musa* spp. ABB Pisang Awak cv. ‘Guangfen No. 1’) fruits at 85–90% plump stage were harvested from a commercial farm in the suburb of Guangzhou City, China. The fruits were pretreated following the method of Song *et al*. [5].

The randomly selected and pretreated fruits were divided into three groups, with 100 fingers per group. Two groups of fruits were directly stored at 7°C and 11°C for 12 days. The third group of fruits were treated with 10^−4^ M ABA [40] and stored at 7°C for 12 days (7°C + ABA). Samples were collected at 0, 3, 6, 9, and 12 days, and their phenotypes were recorded.

Tobacco (*Nicotiana benthamiana*) plants were grown and transformed using the *Agrobacterium tumefaciens*-mediated method [21]. Tomato (*Solanum lycopersicum* cv. ‘Micro-Tom’) plants were transformed and grown following the methods of Song *et al*. [21].

### Measurement of physiological parameters

The CI index of ‘Fenjiao’ banana was evaluated according to Kondo *et al*. [50]. Fruit firmness, ethylene production, and L lightness value were determined according to previous work by Song *et al*. [21]. REC and the MDA content of tomato plants were measured according to the methods of Kong *et al*. [51]. The proline content was measured according to Wang *et al*. [52]. The endogenous ABA content was measured following a method described by Ma *et al*. [53]. The fatty acid and flavonoid contents of ‘Fenjiao’ banana fruits and tomato plants were determined following a method described by Song *et al*. [14] and Chang *et al*. [54], respectively. The cold resistance index of tomato plants was measured according to the method by Wang *et al*. [55].

### Gene expression analysis

Total RNA was extracted, and RT–qPCR was performed according to the methods of Song *et al*. [5]. Tomato *SlUBI* [56] and banana *MaACTIN* [57] were used as the reference for the gene expression analysis in tomato and banana, respectively.

### Dual-luciferase reporter assay

The promoter sequences of 6 fatty acid desaturation genes and 12 flavonoid synthesis-related genes were inserted into the pGreenII 0800-LUC vector and used as reporters. Meanwhile, the full coding sequence of *MaABI5-like* inserted into the pGreenII 62-SK was used as the effector [5]. The reporter and effector plasmids were cotransformed into tobacco (*N. benthamiana*) leaves. Luciferase activity was determined following a method described by Song *et al*. [5]. The LUC/REN ratio was measured using six replicates.

### Yeast one-hybrid assay

The Matchmaker™ Gold Yeast One-Hybrid System (Clontech, Cat. No. 630491) was used in this study for the Y1H assay. The promoters of *MaFAD3-1*, *MaFAD3-4*, *MaFAD3-5*, *MaFAD6-2*, *MaFAD6-3*, *MaPAL-like*, *MaPAL-like1*, *MaC4H-like3*, *Ma4CL-like1*, *Ma4CL-like10*, *MaCHS6-4-like*, and *MaFLS* were inserted into pAbAi as the bait vector. The constructed vectors were linearized and transformed into the Y1H Gold strain. *MaABI5-like* gene integrated into the pGADT7 vector was transferred into the aforementioned bait-reporter yeast strain [21]. The interactions between *MaABI5-like* and the promoters of *MaFAD3-1*, *MaFAD3-4*, *MaFAD3-5*, *MaFAD6-2*, *MaFAD6-3*, *MaPAL-like*, *MaPAL-like1*, *MaC4H-like3*, *Ma4CL-like1*, *Ma4CL-like10*, *MaCHS6-4-like*, and *MaFLS* were analyzed according to Song *et al*. [5].

### Electrophoretic mobility shift assay

The GST-MaABI5-like protein was obtained in our previous study [21]. Probes containing the MaABI5-like binding site (ABRE/G-box) obtained from the promoter regions of the fatty acid desaturation genes and flavonoid synthesis-related genes were labeled using the PierceTM Biotin 3′ End DNA Labeling Kit (Thermo Scientific, Cat. No. 89818). Unlabeled DNA fragments and the ABRE/G-box base changed to A were used as the competitor and the mutant. EMSA was conducted as Xiao *et al*. [58] described using the Light Shift Chemiluminescent EMSA Kit (Thermo Scientific, Cat. No. 20148).

### Chromatin immunoprecipitation–qPCR analysis

The ChIP–qPCR assay was conducted [21]. The chromatin extracts were isolated from banana fruit pulp and cross-linked with 1% (v/v) formaldehyde. The chromatin was prepared at an average length of 500 bp by sonication and immunoprecipitated with MaABI5-like polyclonal antibody; IgG was set as the control. The DNA immunoprecipitated with MaABI5-like polyclonal antibody and IgG was measured by RT–qPCR.

### Transient overexpression analysis in ‘Fenjiao’ banana fruit

The MaABI5-like-pMDC32 construct was transformed into the *A. tumefaciens* strain and transiently overexpressed in ‘Fenjiao’ banana fruit [21]. After infection with the empty vector and the overexpression construct, the fruits were stored at 7°C for 10 days and transferred to 22°C for 4 days. The fruit chilling index, color index, fruit and pulp firmness, ethylene production, and gene expression were determined on days 0, 4, 7, 10, 10 + 2 (10 days of storage under 7°C and 2 days of storage under 22°C), and 10 + 4 (10 days of storage under 7°C and 4 days of storage under 22°C).

### Virus-induced gene silencing in ‘Fenjiao’ banana fruit

The 300 bp gene fragment of *MaABI5-like* was cloned in pTRV2 vector. Then, the MaABI5-like-pTRV2 and pTRV2 constructs were transformed into the *A. tumefaciens* strain. ‘Fenjiao’ banana fruits were pretreated as described above, and divided randomly into four groups. ‘Fenjiao’ banana fruits of groups 1 and 2 were infected with the pTRV2-empty and MaABI5-like-pTRV2 *A. tumefaciens* strain with pTRV1 *A. tumefaciens* at a 1:3 ratio, respectively. For groups 3 and 4, the fruits were treated with 10^−4^ M ABA as described above and infected with pTRV2 (ABA+pTRV2) and MaABI5-like-pTRV2 (ABA+MaABI5-like-PTRV2) *A. tumefaciens* strain with pTRV1 *A. tumefaciens* at a 1:3 ratio, respectively. Then, all group samples were stored at 7°C for 12 days. The fruit chilling index, L, REC, and gene expression levels were determined on days 0, 3, 7, 9, and 12.

### Temperature treatment of tomato plants and fruits

The *MaABI5-like-*overexpressing tomato plants were obtained in our previous study [21]. Three transgenic lines (T2; OA1, OA2, and OA3) were used for the experiment. Four-week-old transgenic plants and WT plants were subjected to chilling stress (4°C) for 5 days in a growth chamber [16 h light (22°C)/8 h dark cycle]. Four plants were maintained per replicate of each tomato genotype, and three biological repeats were maintained per treatment. For the CI test, the WT, OA1, OA2, and OA3 tomato fruits were harvested at the mature red stage and washed with distilled water. These fruits were stored at 4°C for 28 days.

### Data analysis

Data are presented as mean ± standard deviation (SD) using three or six independent biological replicates. ANOVA determined the statistical difference between the mean values, following Duncan’s multiple range test. All primers used in the present work are listed in Supplementary Data Table S1.

## Acknowledgements

This research was supported by the earmarked fund for CARS (grant CARS-31), the National Natural Science Foundation of China (grants 31372112), Guangdong Provincial Special Fund For Modern Agriculture Industry Technology Innovation Teams (grant 2022KJ109), Pearl River Talent Program for Young Talent (grant 2017GC010321), the National Key Research and Development Program (grant 2016YFD0400103), and the Young Innovative Talents Projects in Ordinary Colleges and Universities in Guangdong Province (grant 2021KQNCX005).

## Author contributions

X.Z., W.C., and Xu.L. conceived and designed the experiment. Z.S., Xi.L., H.C., and L.W. performed the experiments. X.Z. and Z.S. carried out the analysis. Z.S. and X.Z. wrote the manuscript. Xu.L., W.C., X.P., Y.H. and W.L. revised the manuscript. All the authors read and approved the final manuscript.

## Data availability

All data that support the findings of this study are available from a corresponding author upon reasonable request.

## Conflict of interest

The authors declare no conflicts of interest.

## Supplementary data

Supplementary data is available at *Horticulture Research* online.

## Supplementary Material

Web_Material_uhac130
