# Supplementary material for: Role of MaABI5-like in abscisic acid-induced cold tolerance of ‘Fenjiao’ banana fruit
Source: Hortic Res. 2022 Jun 3;9:uhac130. doi: 10.1093/hr/uhac130 (PMC10021067; doi:10.1093/hr/uhac130)
Supplement: Web_Material_uhac130 [file web_material_uhac130.zip › Supplementary data.pdf]

Supplementary data

Role of MaABI5-like gene in abscisic acid-induced cold tolerance of 'Fenjiao' banana fruit

Zunyang Song[1,2], Xiuhua Lai[1], Hangcong Chen[1], Lihua Wang[1], Xuequn Pang[1], Yanwei Hao[1], Wangjin Lu[1], Weixin Chen[1], Xiaoyang Zhu[1]*, Xueping Li[1]*

[1]Guangdong Provincial Key Laboratory of Postharvest Science of Fruits and Vegetables/Engineering Research Center for Postharvest Technology of Horticultural Crops in South China, Ministry of Education, College of Horticulture, College of Horticulture, South China Agricultural University, Guangzhou, Guangdong, 510642, CHINA; [2]Key Laboratory of Food Processing Technology and Quality Control in Shandong Province, College of Food Science and Engineering, Shandong Agricultural University, Tai'an, 271018, China

* Corresponding: lxp88@scau.edu.cn (X. Li); xiaoyang_zhu@scau.edu.cn (X. Zhu); Tel.: +86-20-38294892 (X. Zhu); Fax: +86 020 85288280 (X. Zhu)

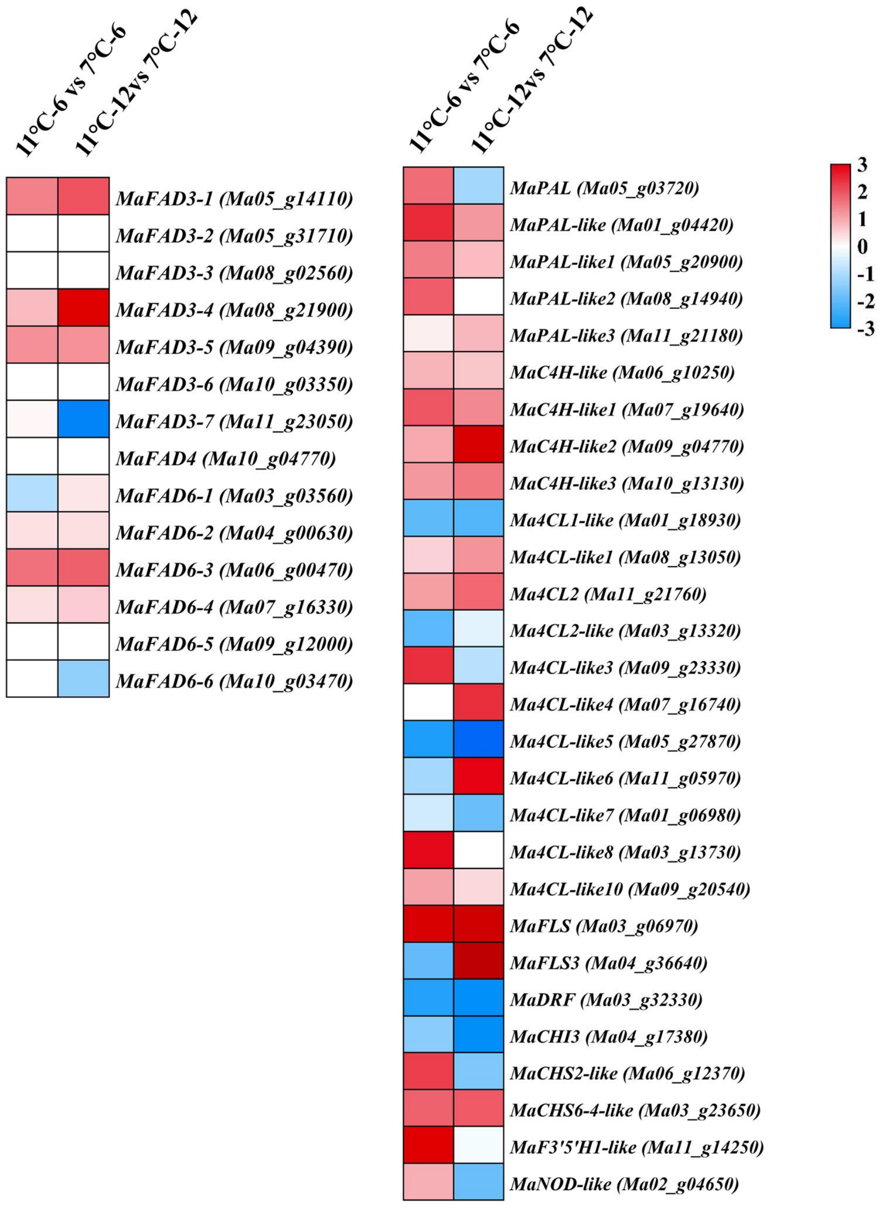

Fig. S1 RNA-Seq analysis showed that genes in fatty acid desaturation and flavonoid synthesis pathway differentially expressed under 11°C and 7°C storage. The expression image was generated using Tb-tools software.

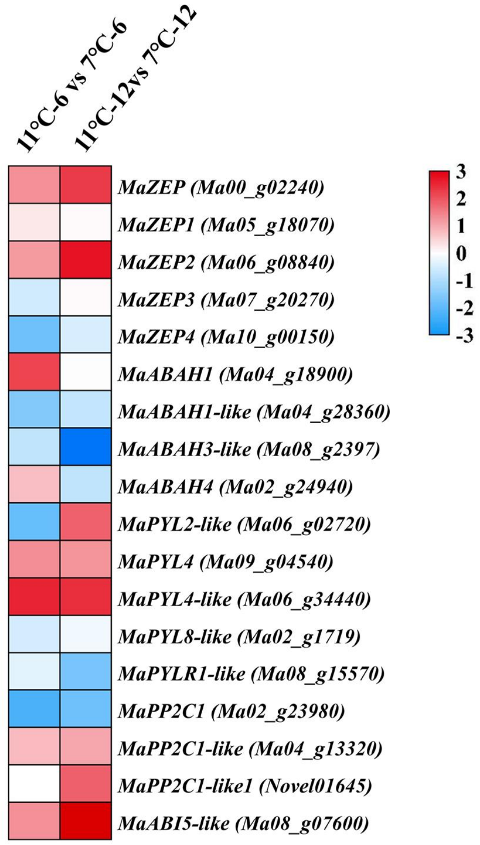

Fig. S2 RNA-Seq analysis showed that genes in ABA synthesis and signal transduction pathway differentially expressed under 11°C and 7°C storage. The heatmap was generated using Tb-tools software.

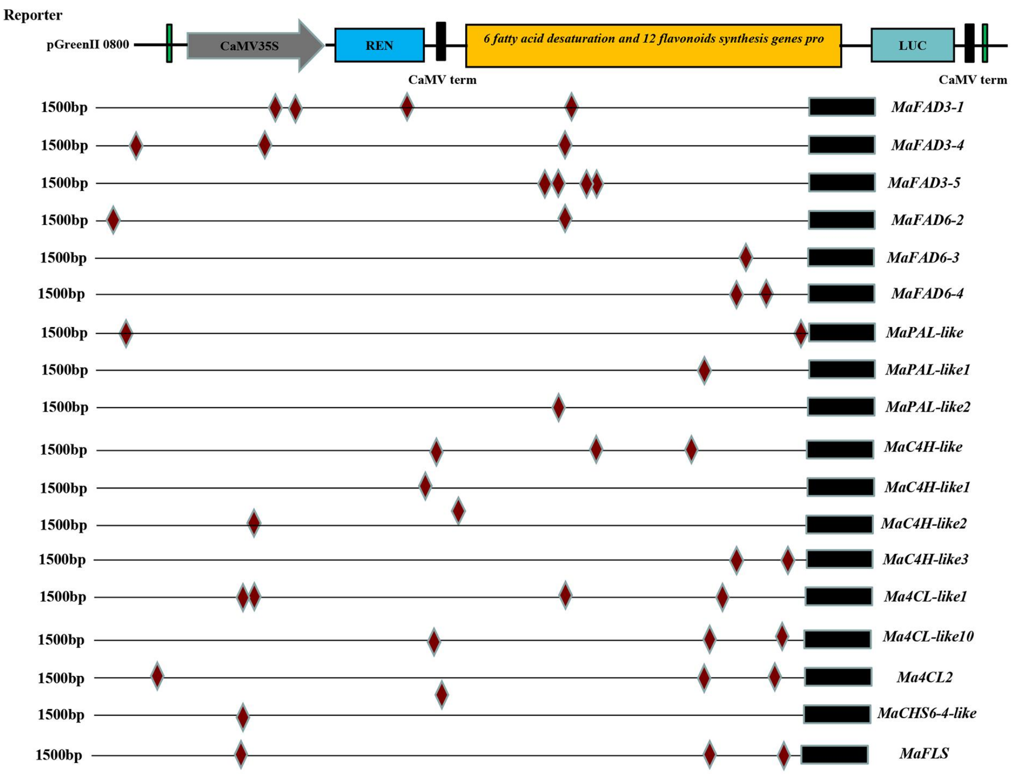

Fig. S3 The location of ABRE/G-box motifs in 6 fatty acid desaturation and 12 flavonoid synthesis genes promoters. The diamonds indicated the ABRE/G-box motifs.

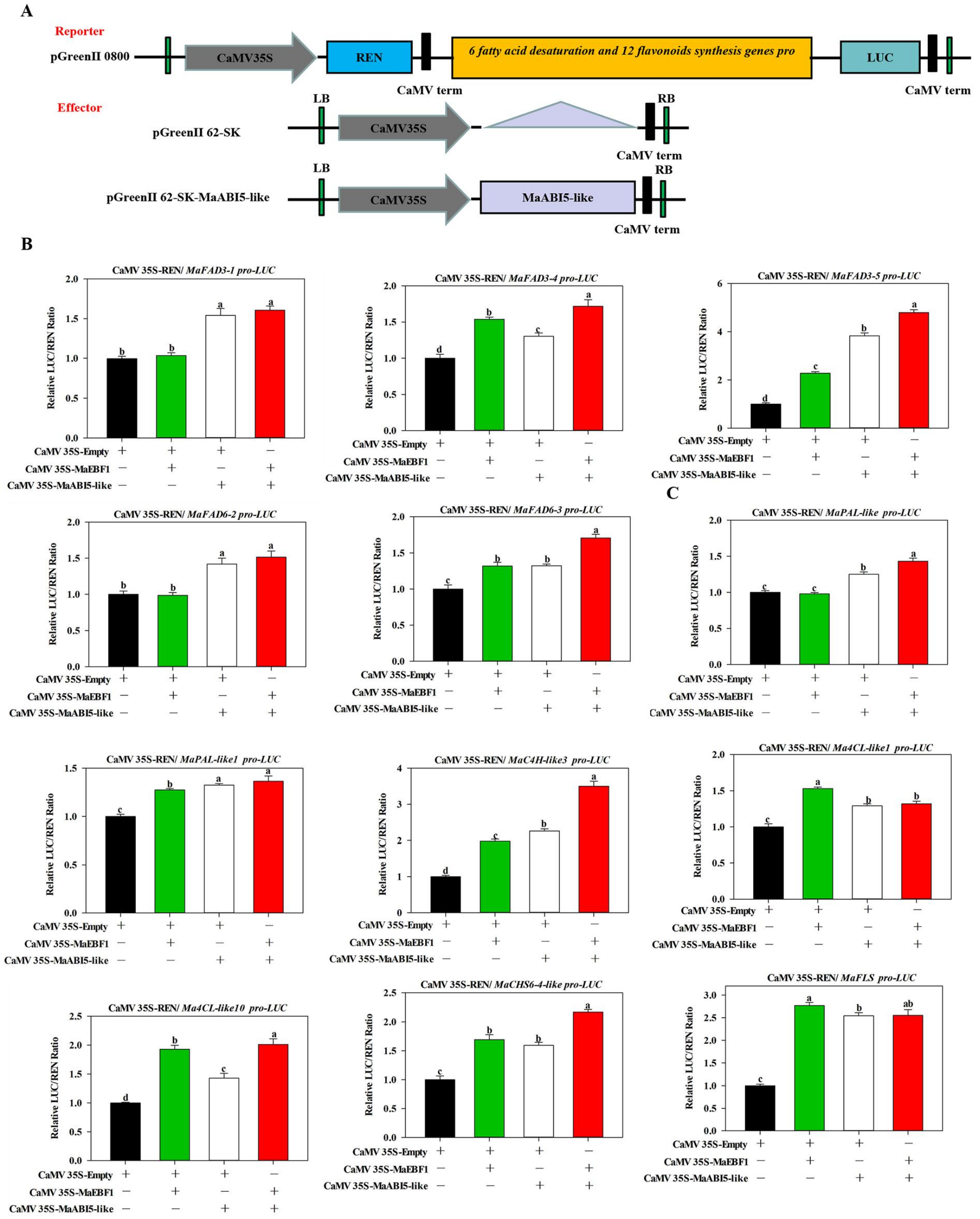

Fig. S4 The effects of interaction between MaEBF1 and MaABI5-like on the activities of promoter of genes related to fatty acid desaturation and flavonoid synthesis. a Diagrams of the reporter and effector constructs used in the dual-luciferase reporter assay. **b**, **c** Transcriptional activity of MaABI5-like, MaEBF1 and MaABI5-like + MaEBF1 on the promoter activities of 5 fatty acid desaturation (**b**) and 7 flavonoid synthesis (**c**) genes. Different letters indicate significantly different values ($P < 0.05$). '+' indicated present, and '-' indicated absent.

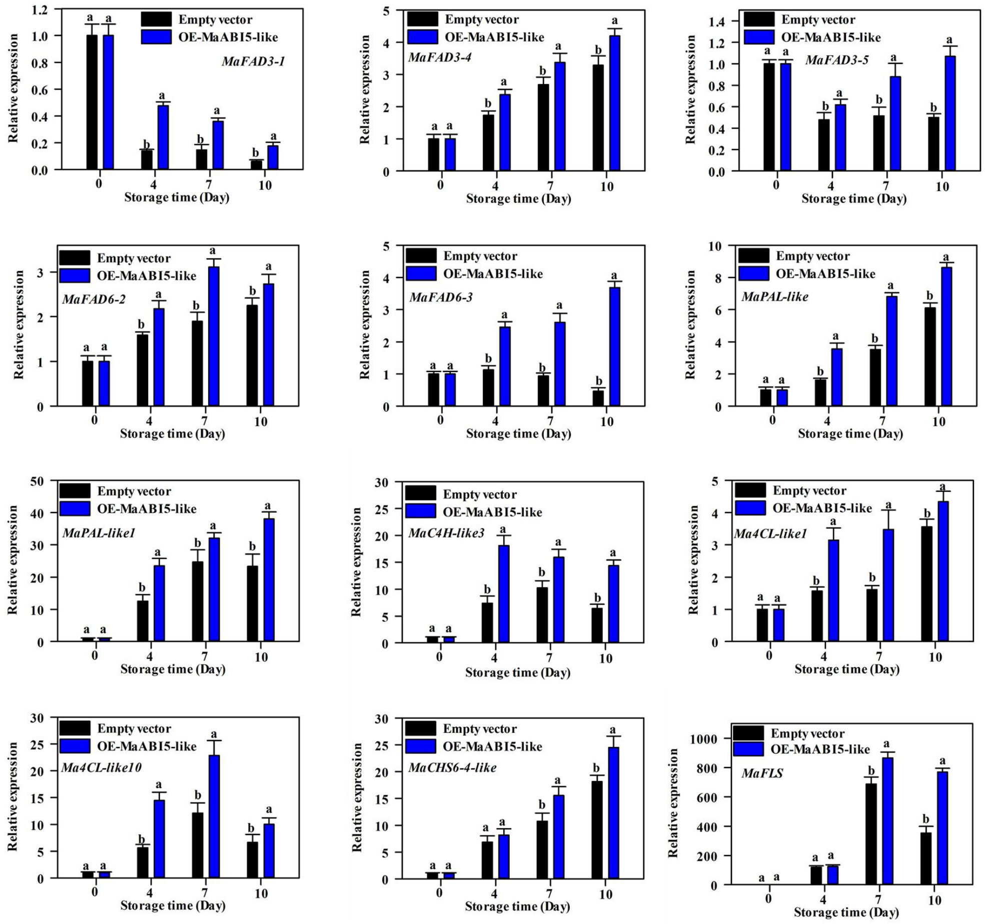

Fig. S5 The expression profiles of genes related to fatty acid desaturation and flavonoid synthesis in transient over-expression of *MaABI5-like* of 'Fenjiao' banana. The transcription of each gene at different points are relative to 0 d set as 1. Different letters indicate significantly different values ($P < 0.05$).

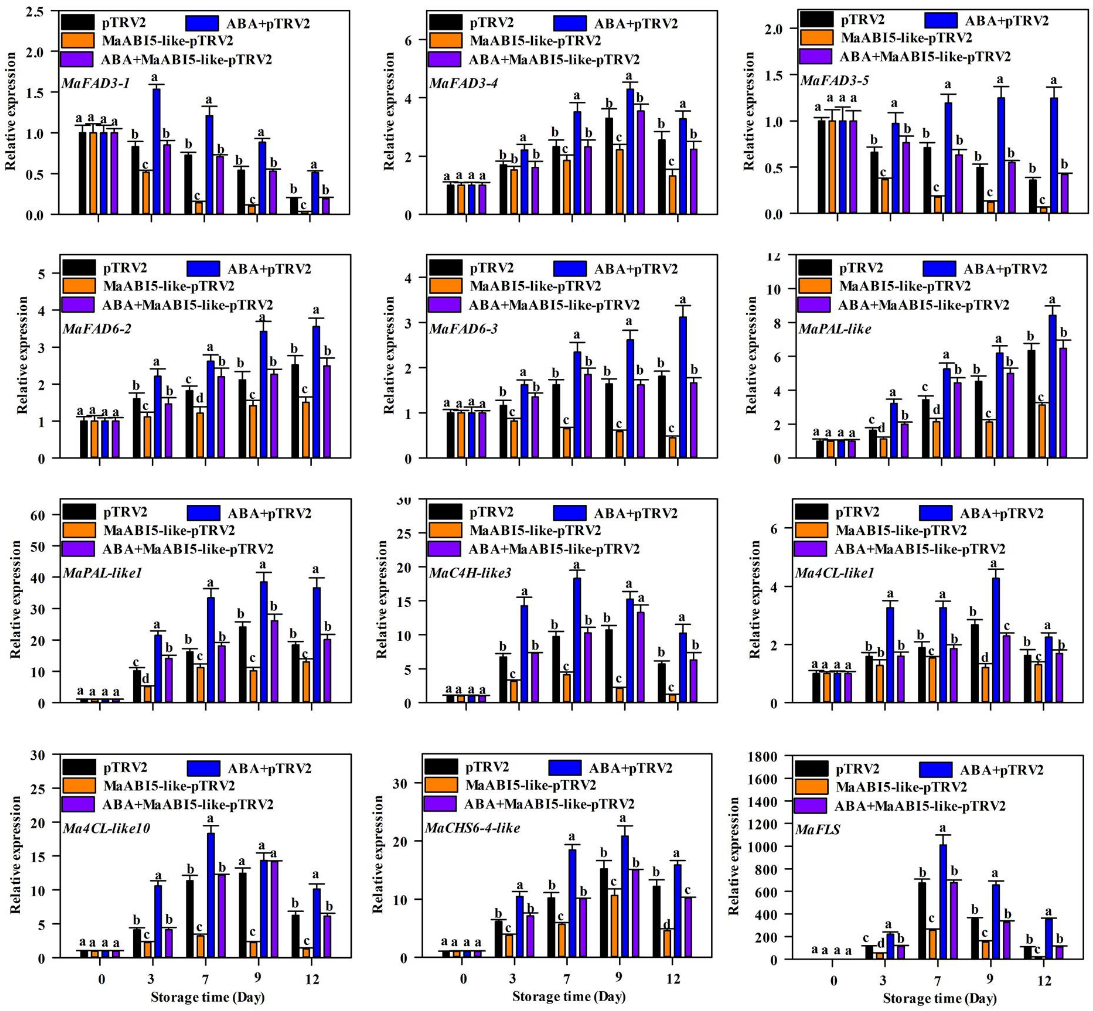

Fig. S6 The expression profiles of genes related to fatty acid desaturation and flavonoid synthesis in transient silencing of *MaABI5-like* of 'Fenjiao' banana. The transcription of each gene at different points are relative to 0 d, which set as 1. Different letters indicate significantly different values ($P < 0.05$)

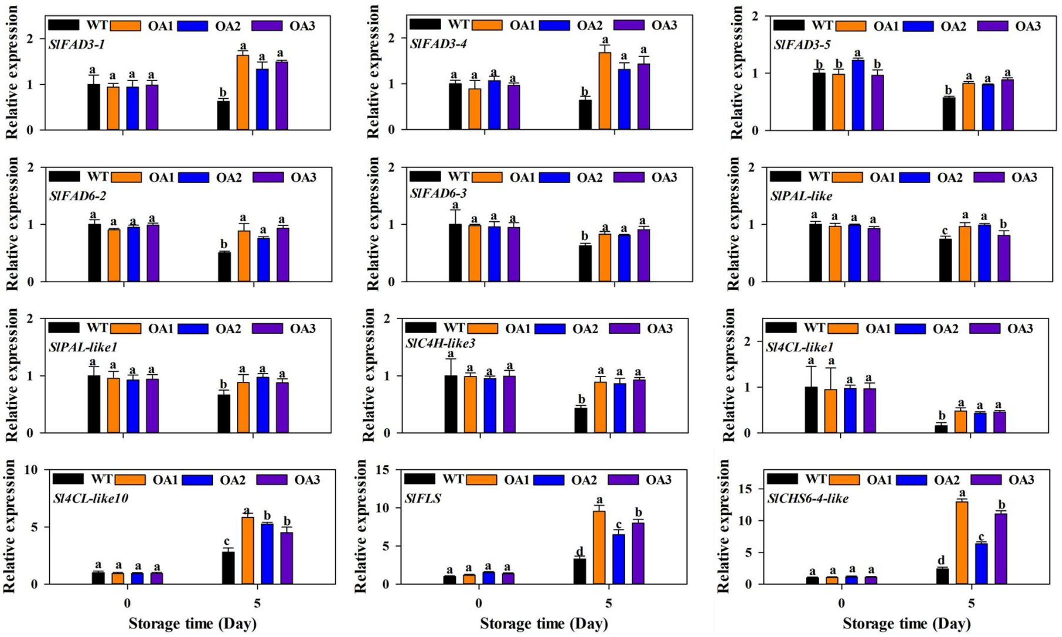

Fig. S7 The expression profiles of genes related to fatty acid desaturation and flavonoid synthesis in over-expression of *MaABI5-like* of tomato. The transcription of each gene at different days are relative to 0 d (WT plant) set as 1. Different letters indicate significantly different values ($P < 0.05$).

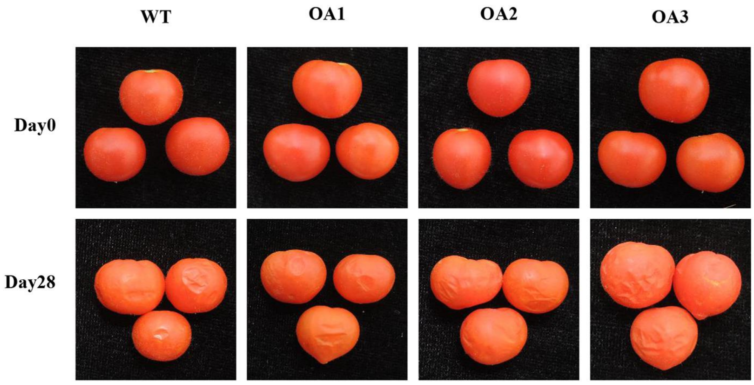

Fig. S8 The cold tolerance phenotype of tomato fruit. Three over-expression of *MaABI5-like* lines (OA1, OA2 and OA3) and WT were harvested at the full-ripening stage and then stored under 4°C for 28 days.

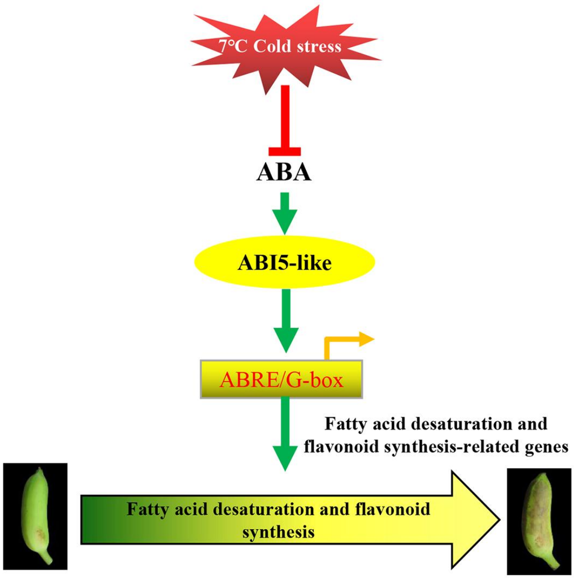

Fig. S9 A proposed working model of MaABI5-like regulating fatty acid desaturation and flavonoid synthesis and fruit chilling injury caused by cold stress.

Table S1. Primers used in present study.

Experiment	Gene ID	Gene	Forward primer (5'-3')
RT-qPCR		MaACTIN-F	CTCCTATGTTGCTCGCTTATG
		MaACTIN-R	GGCTACTACTTCGGTTCTTTC
	XM_009413679.2	MaABI5-like-F	TGAGGCAGAACACCATCTATTC
		MaABI5-like-R	GGTGAGGAACTCATCCATGTT
	Ma05_g14110	MaFAD3-1-F	CGTACCACACTTGGTCTTCAT
		MaFAD3-1-R	CTCCTCGCAGATAGTTCCATTC
	Ma08_g21900	MaFAD3-4-F	CTTGCCTAATGGGTCCTCTTC
		MaFAD3-4-R	ACTCCATTCCTTTCCACGATAC
	Ma09_g04390	MaFAD3-5-F	TGGTATCGTGGAAAGGAATGG
		MaFAD3-5-R	GTGCCAATATCGTGGTGGATA
	Ma04_g00630	MaFAD6-2-F	CACTGGGCCAGATCAAGAA
		MaFAD6-2-R	GAAGAGGGAGGCGAAGAAC
	Ma06_g00470	MaFAD6-3-F	GCGCTTCTCCCTTCTTCTTTAT
		MaFAD6-3-R	TCCTCTGCCCGTTTCATTTC
	Ma07_g16330	MaFAD6-4-F	CGTGGCTGGGATATCACTTT
		MaFAD6-4-R	TGAGCAGCATTCCACTCTTC
	Ma01_g04420	MaPAL-like-F	CAGGTCTTACCCGCTTTACA
		MaPAL-like-R	AACACCTTGTCGAACTCCTC
	Ma05_g20900	MaPAL-like1-F	GGAGTTCGACAAGGTGTTCAT
		MaPAL-like1-R	CGTTCCATTCCTTCAAGCATTC
	Ma08_g14940	MaPAL-like2-F	CTTGGAGGAGAACCTCAAGAAG
		MaPAL-like2-R	CTCCCTCACAAACCGATACAA
	Ma06_g10250	MaC4H-like-F	ACAACTACGGCGACTTCATC
		MaC4H-like-R	GTTCCTCCATCTTCTTCCTCTTC
	Ma07_g19640	MaC4H-like1-F	ACAACTACGGCGACTTCATC
		MaC4H-like1-R	CAGTTGGTCCATCGTCTTCTT
	Ma09_g04770	MaC4H-like2-F	GGCTGGTGCTGATGATGT
		MaC4H-like2-R	TGATGGCCTTGAGCTTGTT
	Ma10_g13130	MaC4H-like3-F	CTCAAGAAGTGTCAAGACCTCAA
		MaC4H-like3-R	CGATCCCAATTCGTCCATCTT
	Ma08_g13050	Ma4CL-like1-F	CTGATGTCGTCCAGGGTTATG
		Ma4CL-like1-R	AACTTCCGACGCCAGTATTC
	Ma09_g20540	Ma4CL-like10-F	GAGCTTGTGATCGTGTTCCT
		Ma4CL-like10-R	ACTCCGAGTCAGAGAGGTAAA
	Ma11_g21760	Ma4CL2-F	CCACATATTCGCCCTGAACT
		Ma4CL2-R	ACACCCTCTGCCTCTGTAT
	Ma03_g23650	MaCHS6-4-like-F	CTCAATCGGCGAGCAAGAT
		MaCHS6-4-like-R	CGATGACGTTGGGAGGATTAG
	Ma03_g06970	MaFLS-F	GGACTTCCTCTTCCACAACATC
		MaFLS-R	CTCCACCAATCCCACCAAAT
	Ma04_g36640	MaFLS3-F	GACGTAGATGGTGAGGCTATTC
		MaFLS3-R	TCGTCATGTACCTCTCTCTCTC
		SlUBI-F	GCCGACTACAACATCCAGAAGG
		SlUBI-R	TGCAACACAGCGAGCTTAACC
	XM_015223673.2	SlFAD3-1-F	TTCCCTCAAATCCCACACTATC
		SlFAD3-1-R	CCTGACTTCTTTGGCTCCTTAT

	XM_015223673.2	SlFAD3-4-F	ACAGAGGAGAGGAATGGAGTTA
		SlFAD3-4-R	GTGGGATTTGAGGGAAGAGATG
	NM_001247663.1	SlFAD3-5-F	TTCCCTCAAATCCCACACTATC
		SlFAD3-5-R	CCTGACTTCTTTGGCTCCTTAT
	XM_004228619.4	SlFAD6-2-F	ACTTCCACCTCATTCCATCAC
		SlFAD6-2-R	CGTGGGCATTAACCCAAATTC
	XM_004228617.4	SlFAD6-3-F	ACTTCCACCTCATTCCATCAC
		SlFAD6-3-R	CGTGGGCATTAACCCAAATTC
	NM_001320040.1	SlPAL-like-F	GGGCTTAATCTCAGCAAGGAA
		SlPAL-like-R	TCCAAATGCCTCAAGTCGATAG
	XM_004246601.3	SlPAL-like1-F	CCCTTGCATCTATTGGGAAATTG
		SlPAL-like1-R	ATTCCTTCCTGCTGTGAGATTAG
	XM_004240074.4	SlC4H-like3-F	GGGCTTGTGATAGCCAAACTA
		SlC4H-like3-R	CAAGCTAAACTGCCCTCCTT
	XM_004242742.3	Sl4CL-like1-F	CGCCGGAGAAATTTGCATTAG
		Sl4CL-like1-R	GTATCCAATGTCACCCGTATGT
	XM_004234347.4	Sl4CL-like10-F	GGGTACAAGAACAATCCAGAGG
		Sl4CL-like10-R	CTGCCAACCAAATGCAAGTATC
	NM_001247104.2	SlCHS6-4-like-F	AGAACAACAAGGGTGCTAGAG
		SlCHS6-4-like-R	GGCCTACCAAGCTATCTAAGTG
	XM_004250281.4	SlFLS-F	GCTGGTGGTGAAGACATAGTT
		SlFLS-R	TTGGGACAAGAAGGGTGATATG
Transient expression assay		MaABI5-like-SK-F	CGCTCTAGAACTAGTGGATCCATGGCGTCCCTGTCGGAGAGCAGG
		MaABI5-like-SK-R	GATAAGCTTGATATCGAATTCTCACCAAGTGCTGCTGTTGCAGC
		MaFAD3-1-0800--F	CTATAGGGCGAATTGGGTACCAGCGAGCAGACCACCTCATC
		MaFAD3-1-0800-R	TGTTTTTGGCGTCTTCCATGGAGACCAGGGCCAGATGTAG
		MaFAD3-4-0800-F	CTATAGGGCGAATTGGGTACCATCATCAATCACTGGACCGAC
		MaFAD3-4-0800--R	TGTTTTTGGCGTCTTCCATGGCGTGAACCGAAGCTTCTG
		MaFAD3-5-0800-F	CTATAGGGCGAATTGGGTACCTGTGATCAAGGATGATGATGC
		MaFAD3-5-0800-R	TGTTTTTGGCGTCTTCCATGGAGGAGAAGGCGGAGATCAG
		MaFAD6-2-0800-F	CTATAGGGCGAATTGGGTACCTTCTCTCTCAGGTACGTG
		MaFAD6-2-0800-R	TGTTTTTGGCGTCTTCCATGGCTGCTATGGGCTCACCTG
		MaFAD6-3-0800-F	CTATAGGGCGAATTGGGTACCTGGCATATGTCTATCTGAGTC
		MaFAD6-3-0800-R	TGTTTTTGGCGTCTTCCATGGACCCCAAATAACACACTTGC
		MaFAD6-4-0800-F	CTATAGGGCGAATTGGGTACCTAGATGGGCTCCCAACGTG
		MaFAD6-4-0800-R	TGTTTTTGGCGTCTTCCATGGCGTAAGAGAGGTAATAGAC
		MaPAL-like-0800-F	CTATAGGGCGAATTGGGTACCTCTCTCTACTTTCGTGGTAG
		MaPAL-like-0800-R	TGTTTTTGGCGTCTTCCATGGTTCAATACGTGAATAGGTC
		MaPAL-like1-0800-F	CTATAGGGCGAATTGGGTACCAGGTTCCACTTAAGTCATG
		MaPAL-like1-0800-R	TGTTTTTGGCGTCTTCCATGGATAATACTTCAACCACTG
		MaPAL-like2-0800-F	CTATAGGGCGAATTGGGTACCGATCCATGAATTGGATCAGC
		MaPAL-like2-0800-R	TGTTTTTGGCGTCTTCCATGGAGAGCGAGAGAGAAGTGC
		MaC4H-like-0800-F	CTATAGGGCGAATTGGGTACCATCCTTTCAGATGACAGTG
		MaC4H-like-0800-R	TGTTTTTGGCGTCTTCCATGGTTGACTTGTGTTCTGCAG
		MaC4H-like1-0800-F	CTATAGGGCGAATTGGGTACCTGCAGTATAAAGACACTG
		MaC4H-like1-0800-R	TGTTTTTGGCGTCTTCCATGGAGGCGGTGAAAGAGTGACTG
		MaC4H-like2-0800-F	CTATAGGGCGAATTGGGTACCAAATCTTGGGTTGAACTG
		MaC4H-like2-0800-R	TGTTTTTGGCGTCTTCCATGGAGAAGAGCAAGTGTCCGTC
		MaC4H-like3-0800-F	CTATAGGGCGAATTGGGTACCTCACCTAAACCACTACGAC
		MaC4H-like3-0800-R	TGTTTTTGGCGTCTTCCATGGTGAAGATATCGAACACCACG
		Ma4CL-like1-0800-F	CTATAGGGCGAATTGGGTACCTGAAATTCGACATGATCATC
		Ma4CL-like1-0800-R	TGTTTTTGGCGTCTTCCATGGAGGATGAGATCTTTGAGC
		Ma4CL-like10-0800-F	CTATAGGGCGAATTGGGTACCAATGGTTTAGTGGAGACG

	Ma4CL-like10-0800-R	TGTTTTTGGCGTCTTCCATGGAGCAGAGAAAACAAAGCTAC
	Ma4CL2-0800-F	CTATAGGGCGAATTGGGTACCATGACCAAAGATTAGATGC
	Ma4CL2-0800-R	TGTTTTTGGCGTCTTCCATGGTGTTGCTACTCTTTCTGCAG
	MaCHS6-4-like-0800-F	CTATAGGGCGAATTGGGTACCTCAATTTGGCCTTGTGATC
	MaCHS6-4-like-0800-R	TGTTTTTGGCGTCTTCCATGGTTGTACGAGTCAGCGACTG
	MaFLS-0800-F	CTATAGGGCGAATTGGGTACCAGAGTGTCAGGTAAGACTG
	MaFLS-0800-R	TGTTTTTGGCGTCTTCCATGGTGTTGTTGGTAGCAGTAC
	MaFLS3-0800-F	CTATAGGGCGAATTGGGTACCTCATGCTCTATGAAGCTCTAC
	MaFLS3-0800-R	TGTTTTTGGCGTCTTCCATGGTGGCTCAACGGAGTTAAGTCG
Y1H	MaFAD3-1-PAbAi-F	TTGAATTCGAGCTCGGTACCTTTTACGTGCTCATTTTACGTGCTCATTTTACGTGCTCA
	MaFAD3-1-PAbAi-R	ATGCCTCGAGGTCGACTGAGCACGTAAAATGAGCACGTAAAATGAGCACGTAAAA
	MaFAD3-4-PAbAi-F	TTGAATTCGAGCTCGGTACCGTAGCACGACACAGTAGCACGACACAGTAGCACGACACA
	MaFAD3-4-PAbAi-R	ATGCCTCGAGGTCGACTGTGTCGTGCTACTGTGTCGTGCTACTGTGTCGTGCTAC
	MaFAD3-5-PAbAi-F	TTGAATTCGAGCTCGGTACCTGCTACGTGTAAGTGCTACGTGTAAGTGCTACGTGTAAG
	MaFAD3-5-PAbAi-R	ATGCCTCGAGGTCGACCTTACACGTAGCACTTACACGTAGCACTTACACGTAGCA
	MaFAD6-2-PAbAi-F	TTGAATTCGAGCTCGGTACCCGTCATACGTGGGCGTCATACGTGGGCGTCATACGTGGG
	MaFAD6-2-PAbAi-R	ATGCCTCGAGGTCGACCCCACGTATGACGCCCACGTATGACGCCCACGTATGACG
	MaFAD6-3-PAbAi-F	TTGAATTCGAGCTCGGTACCACGATACGTGCAGACGATACGTGCAGACGATACGTGCAG
	MaFAD6-3-PAbAi-R	ATGCCTCGAGGTCGACCTGCACGTATCGTCTGCACGTATCGTCTGCACGTATCGT
	MaPAL-like-PAbAi-F	TTGAATTCGAGCTCGGTACCCGATCACGTAAATCGATCACGTAAATCGATCACGTAAAT
	MaPAL-like-PAbAi-R	ATGCCTCGAGGTCGACATTTACGTGATCGATTTACGTGATCGATTTACGTGATCG
	MaPAL-like1-PAbAi-F	TTGAATTCGAGCTCGGTACCATGAACGTGGAAAATGAACGTGGAAAATGAACGTGGAAA
	MaPAL-like1-PAbAi-R	ATGCCTCGAGGTCGACTTTCCACGTTCATTTTCCACGTTCATTTTCCACGTTCAT
	MaC4H-like3-PAbAi-F	TTGAATTCGAGCTCGGTACCCGCAACGTGGTGTCGCAACGTGGTGTCGCAACGTGGTGT
	MaC4H-like3-ABI5-R	ATGCCTCGAGGTCGACACACCACGTTGCGACACCACGTTGCGACACCACGTTGCG
	Ma4CL-like1-PAbAi-F	TTGAATTCGAGCTCGGTACCTTCACGTGATCAGTTCACGTGATCAGTTCACGTGATCAG
	Ma4CL-like1-PAbAi-R	ATGCCTCGAGGTCGACCTGATCACGTGAACTGATCACGTGAACTGATCACGTGAA
	Ma4CL-like10-PAbAi-F	TTGAATTCGAGCTCGGTACCCCTACACGACAAGCCTACACGACAAGCCTACACGACAAG
	Ma4CL-like10-PAbAi-R	ATGCCTCGAGGTCGACCTTGTCGTGTAGGCTTGTCGTGTAGGCTTGTCGTGTAGG
	MaCHS6-4-like-PAbAi-F	TTGAATTCGAGCTCGGTACCAGCAACGTGCACAAGCAACGTGCACAAGCAACGTGCACA
	MaCHS6-4-like-PAbAi-R	ATGCCTCGAGGTCGACTGTGCACGTTGCTTGTGCACGTTGCTTGTGCACGTTGCT
	MaFLS-PAbAi-F	TTGAATTCGAGCTCGGTACCTGGACACGACAGCTGGACACGACAGCTGGACACGACAGC
	MaFLS-PAbAi-R	ATGCCTCGAGGTCGACGCTGTCGTGTCCAGCTGTCGTGTCCAGCTGTCGTGTCCA
EMSA	MaFAD3-1-probe-F	AGTAAGCTTTCCTACACCTTTTTACGTGCTCAAATTATATACGGGAAA
	MaFAD3-1-probe-R	TTTCCCGTATATAATTTGAGCACGTAAAAAGGTGTAGGAAAGCTTACT
	MaFAD3-4--probeF	CAATGGTCCACGCCATTTCCCACGTGCCGACGCAGTCAACCCCCGTGT
	MaFAD3-4-probe-R	ACACGGGGGTTGACTGCGTCGGCACGTGGGAAATGGCGTGGACCATTG
	MaFAD3-5--probeF	TGCCACAAAGGTCTCTCTGCTACGTGTAAGCTGCTGATAGGTCGGT
	MaFAD3-5-probe-R	ACCGACCTATCAGCAGCTTACACGTAGCAGAGAGACCTTTGTGGCA
	MaFAD6-2--probeF	GAATCTCCAGGCGGGGTCGGCGTCATACGTGGGACGCGAAGTGGTCCG
	MaFAD6-2-probe-R	CGGACCACTTCGCGTCCCACGTATGACGCCGACCCCGCCTGGAGATTC
	MaFAD6-3--probeF	CACAACGCACGAAGTCAACGATACGTGCAGTATGACAACGACGATAGAT
	MaFAD6-3-probe-R	ATCTATCGTCGTTGTCATACTGCACGTATCGTTGACTTCGTGCGTTGTG
	MaPAL-like-probe-F	ATTTTCTTTTGACTACCACAACGATCACGTAAATTTCTCCCTAGCATTC
	MaPAL-like-probe-R	GAATGCTAGGGAGAAATTTACGTGATCGTTGTGGTAGTCAAAAGAAAAT
	MaPAL-like1-probe-F	ATATGACTCGGGTGTGACATGAACGTGGAAATCACCTTCTACTCGGGA
	MaPAL-like1-probe-R	TCCCGAGTAGAAGGTGATTTCCACGTTCATGTCACACCCGAGTCATAT
	MaC4H-like3-probe-F	TCGAGTTCGGCTCCCGCACCCGCAACGTGGTGTTCGATATCTTCAC
	MaC4H-like3-probe-R	GTGAAGATATCGAACACCACGTTGCGGGTGCGGGAGCCGAACTCGA
	Ma4CL-like1-probe-F	TCGACAAAGTAAAATGTGACTTTTCACGTGATCAGCCCGAAACTTCTG
	Ma4CL-like1-probe-R	CAGAAGTTTCGGGCTGATCACGTGAAAAGTCACATTTTACTTTGTCGA
	Ma4CL-like10-probe-F	CACACCCTTTCCCTTCTCCTCCTACACGACAAGAGAAGCCGCGCACAG
	Ma4CL-like10-probe-R	CTGTGCGCGGCTTCTCTTGTCGTGTAGGAGGAGAAGGGAAAGGGTGTG

	MaCHS6-4-like-probe-F	TGGAACTGATGTGTTCCAAGCAACGTGCACATTGGATGCTCCATCCA
	MaCHS6-4-like-probe-R	TGGATGGAGCATCCAATGTGCACGTTGCTTGGAACACATCAGTTCCA
	MaFLS-probe-F	CATGATTTAGCTGGTGGACACGACAGCAAATCCAACACACAACTGCT
	MaFLS-probe-R	AGCAGTTGTGTGTTGGATTTGCTGTCGTGTCCACCAGCTAAATCATG
ChIP-qPCR	MaFAD3-1-ChIP-F	CTTGTCTCGCTGTGGACATAA
	MaFAD3-1-ChIP-R	GACCATTGGCAGAGAATCCA
	MaFAD3-4-ChIP-F	ACCAAACAAGACGGGAAGAA
	MaFAD3-4-ChIP-R	TGCCGTGTTTCGAAGGAA
	MaFAD3-5-ChIP-F	GAGGAGAGCAGCGTCTTTATAC
	MaFAD3-5-ChIP-R	CGTTGTACCCAACACCACTA
	MaFAD6-2-ChIP-F	AACGGCGGTGCACTAAA
	MaFAD6-2-ChIP-R	CCACACGGGAGTGGAATATG
	MaFAD6-3-ChIP-F	CACAACGCACGAAGTCAA
	MaFAD6-3-ChIP-R	CAATTAACGCAAACTGTCTTGT
	MaPAL-like-ChIP-F	CTCTCTACTTTCGTGGTAGTGTAAG
	MaPAL-like-ChIP-R	GAAATTGACGCTCCATGTTGAC
	MaPAL-like1-ChIP-F	ATGCTTATATGACTCGGGTGTG
	MaPAL-like1-ChIP-R	TCTCTCTCTCTCTTTCCAGGAC
	MaC4H-like3-ChIP-F	GATCGTCGTCTCCAAGCTC
	MaC4H-like3-ChIP-R	CGGTGAGATTGCGGTGATTA
	Ma4CL-like1-ChIP-F	GATCCAAGAATCGAAGGGAGAG
	Ma4CL-like1-ChIP-R	TTCGTTGCTGCGAGAGATT
	Ma4CL-like10-ChIP-F	TTTCCACACCCTTTCCCTTC
	Ma4CL-like10-ChIP-R	GGTCGTTGGTTTGGGTTCATA
	MaCHS6-4-like-ChIP-F	ACCCAAACTGTTGGAGAAGAT
	MaCHS6-4-like-ChIP-R	TGAACCACATAGGCACGATAC
	MaFLS-ChIP-F	AGGTAAGACTGTAAGGTACTGAAA
	MaFLS-ChIP-R	CACGTCTCGCACATCAAG
Overexpression and silencing	MaABI5-like-Pdnor-F	AAAAAGCAGGCTTCATGGCGTCCCTGTCGGAGAGCAGG
	MaABI5-like-Pdnor-R	CAAGAAAGCTGGGTCTCACCAAGTGCTGCTGTTGCAGC
	MaABI5-like-pTRV2-F	TACCGAATTCTCTAGAAGCGGTGGACAAGGTGGT
	MaABI5-like-pTRV2-R	CTTCGGGACATGCCCGGGTCTCTTAGTAGGGGAGCT
